# Overcoming the imatinib-resistant BCR-ABL mutants with new ureidobenzothiazole chemotypes endowed with potent and broad-spectrum anticancer activity

**DOI:** 10.1080/14756366.2023.2189097

**Published:** 2023-03-17

**Authors:** Ashraf K. El-Damasy, Heewon Jin, Jung Woo Park, Hyun Ji Kim, Hanan Khojah, Seon Hee Seo, Ju-Hyeon Lee, Eun-Kyoung Bang, Gyochang Keum

**Affiliations:** a Center for Brain Technology, Brain Science Institute, Korea Institute of Science and Technology (KIST), Seoul, Republic of Korea; b Department of Medicinal Chemistry, Faculty of Pharmacy, Mansoura University, Mansoura, Egypt; c Center for Supercomputing Applications, Div. of National Supercomputing R&D, Korea Institute of Science and Technology Information, Daejeon, Republic of Korea; d Department of Pharmacognosy, College of Pharmacy, Jouf University, Sakaka, Saudi Arabia; e Center for Brain Disorders, Brain Science Institute, Korea Institute of Science and Technology (KIST), Seoul, Republic of Korea; f Division of Bio-Medical Science & Technology, KIST School, Korea University of Science and Technology (UST), Seoul, Republic of Korea

**Keywords:** Ureidobenzothiazoles, BCR-ABL^T315I^, imatinib resistance, CML, anticancer activity

## Abstract

The design of kinase inhibitors targeting the oncogenic kinase BCR-ABL constitutes a promising paradigm for treating chronic myeloid leukaemia (CML). Nevertheless, the efficacy of imatinib, the first FDA-approved targeted therapy for CML, is curbed by the emergence of resistance. Herein, we report the identification of the 2-methoxyphenyl ureidobenzothiazole **AK-HW-90 (2b)** as a potent pan-BCR-ABL inhibitor against imatinib-resistant mutants, particularly T315I. A concise array of six compounds **2a**–**f** was designed based on our previously reported benzothiazole lead **AKE-5l** to improve its BCR-ABL^T315I^ inhibitory activity. Replacing the 6-oxypicolinamide moiety of **AKE-5l** with *o*-methoxyphenyl and changing the propyl spacer with phenyl afforded **2a** and **AK-HW-90 (2b)** with IC_50_ values of 2.0 and 0.65 nM against BCR-ABL^T315I^, respectively. **AK-HW-90** showed superior anticancer potency to imatinib against multiple cancer cells (NCI), including leukaemia K-562. The obtained outcomes offer **AK-HW-90** as a promising candidate for the treatment of CML and other types of cancer.

## Graphical abstract

**Figure:**
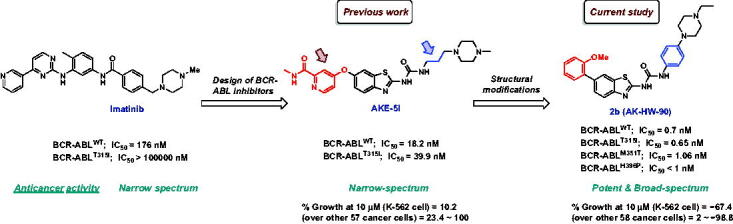

## Introduction

Chronic myeloid leukaemia (CML) is a myeloproliferative neoplasm characterised by excessive production of immature white blood cells and constitutes approximately 15% of leukaemia cases in adults^1^. The fusion oncoprotein product of the Philadelphia chromosome (Ph), Break-point cluster region-Abelson (BCR-ABL), is the critical driver for the pathogenesis of CML and acute lymphoblastic leukaemia (Ph + ALL)^2^. Therefore, the design of BCR-ABL kinase inhibitors offers a legitimate approach for the treatment of CML and ALL.

**Figure 1.**
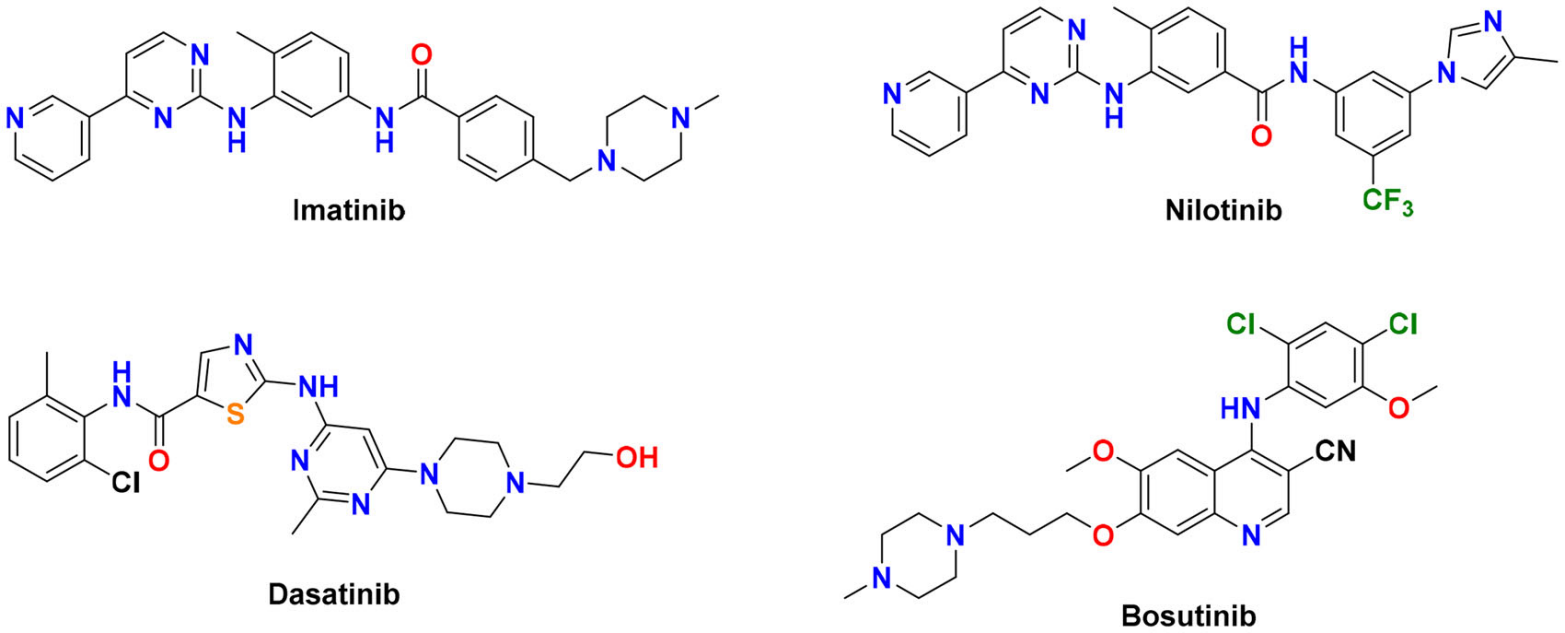
Chemical structures of the first and second-generation BCR-ABL inhibitors.

Imatinib (Gleevec®, (Figure 1)), a diarylamide-4-(pyridin-3-yl)pyrimidine conjugate, is the first FDA-approved BCR-ABL inhibitor for the treatment of CML patients^3^. Despite its initial significant response in most of the CML patients, the clinical efficacy of imatinib was hampered in about 40% of patients due to dose intolerance and the emergence of drug resistance^4^. Point mutations within the BCR-ABL kinase domain represent the most recurrent mechanism of imatinib resistance^5^, where they hinder its proper binding with the target^6^. Among more than 100 different mutations detected in CML patients, the gatekeeper T315I mutation is the most refractory form, which emerges in more than 20% of CML patients^7^. Second-generation BCR-ABL inhibitors, nilotinib (Tasigna®), dasatinib (Sprycel®), and bosutinib (Bosulif®) (Figure 1), have been approved to treat CML adult patients with acquired resistance to imatinib^8–10^. However, these second-line drugs failed to inhibit several imatinib-resistant mutations, including the T315I form^11^.

**Figure 2.**
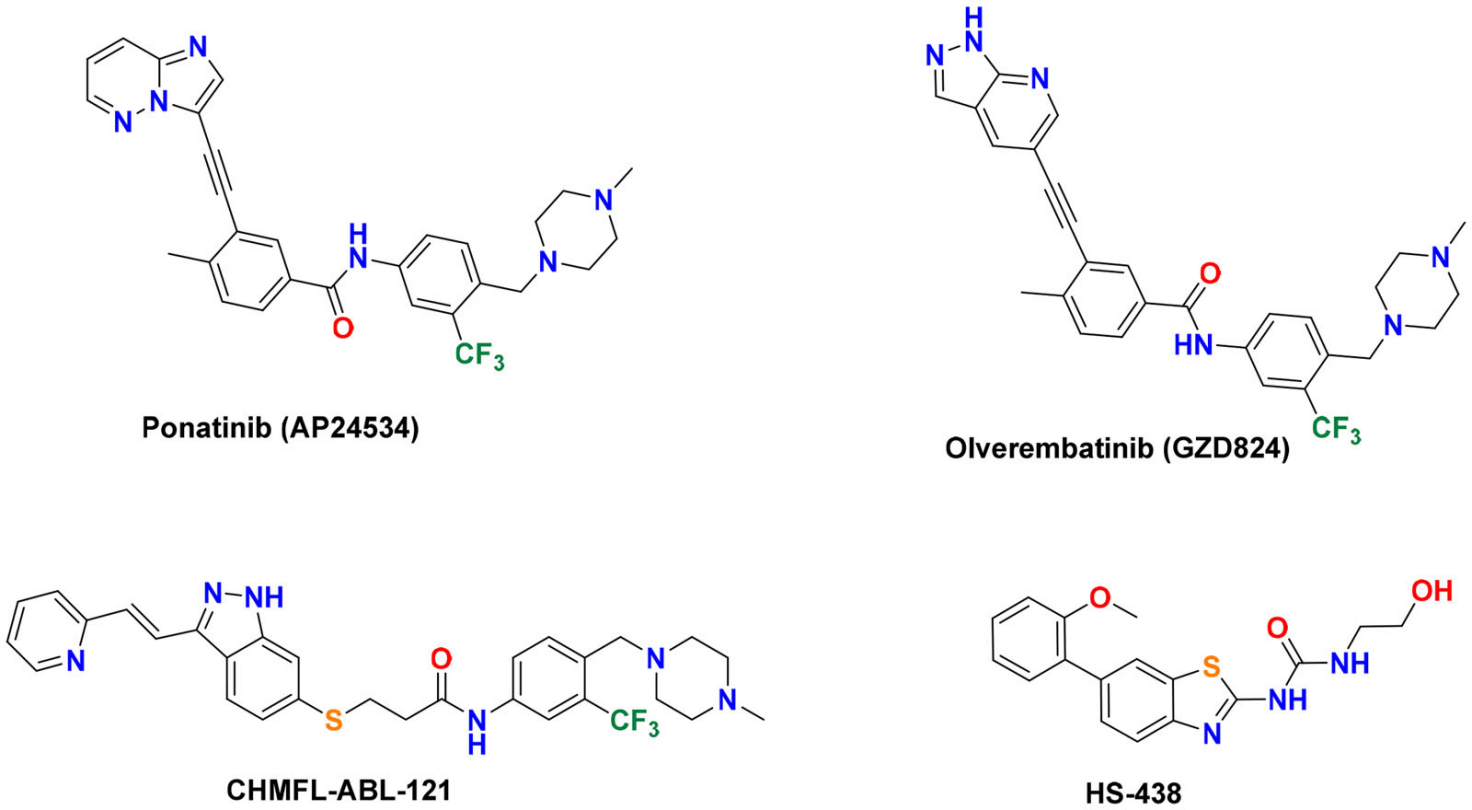
Chemical structures of ponatinib and other potent BCR-ABL^T315I ^inhibitors.

In 2012, the multikinase inhibitor ponatinib (Iclusig®, Figure 2) received FDA approval as the third-generation ABL inhibitor for the treatment of patients with CML and Ph + ALL^12^. It demonstrated excellent potency against imatinib-clinically resistant ABL mutants, including T315I and other forms like Q252H and H396P^13^^,^^14^. However, the serious vascular/cardiotoxic effects associated with ponatinib restricted its use for these tumours harbouring T315I mutant. Ponatinib cardiotoxicity is thought to arise from its multikinase inhibitory mode and synchronous inhibition of kinases that are important for cardiovascular function^15^. Therefore, a number of structurally assorted BCR-ABL^T315I^ inhibitors with improved selectivity/safety have been developed (Figure 2). Olverembatinib (GZD824), a pyrazolopyridine-diarylamide conjugate, was developed from ponatinib as a potent BCR-ABL^T315I^ inhibitor with a favourable safety profile^16^. The indazole derivative CHMFL-ABL-121 is a type-II inhibitor derived from axitinib with an IC_50_ value of 0.2 nM against BCR-ABL^T315I^^17^. The ureidobenzothiazole HS-438 was discovered by Park *et al.* as a pico-molar BCR-ABL^T315I^ inhibitor, which potently inhibited the proliferation of imatinib-resistant Ba/F3^T315I^ cells^18^.

**Figure 3.**
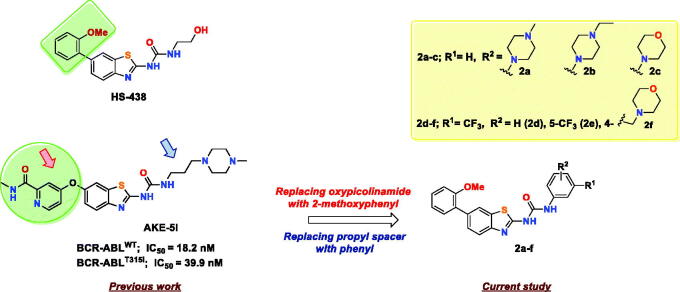
Rational design of the target compounds.

In a previous study, our group reported a series of benzothiazoles featuring picolinamide moiety as BCR-ABL inhibitors^19^. In this set, compound **AKE-5l** (Figure 3) was identified as the most potent BCR-ABL inhibitor with IC_50_ values of 18.2 and 39.9 nM against wild BCR-ABL and its T315I mutant, respectively. Moreover, **AKE-5l** showed selective anti-leukemic activity against only the K-562 cell line out of 58 tested cancer cells. In continuation of our ongoing efforts to identify new chemical entities as potent anticancer kinase inhibitors,^20–25^ we herein aimed at further optimisation of **AKE-5l** activity. Based on the exceptional potency of HS-438 towards BCR-ABL^T315I^ (IC_50_ = 0.064 nM), it was evident that installing 2-methoxyphenyl motif directly at C-6 of benzothiazole is optimal for activity than 6-oxypicolinamide. Therefore, in the current study, we replaced the picolinamide ether of **AKE-5l** with 2-methoxyphenyl moiety, while conserving the ureidobenzothiazole scaffold (Figure 3). In addition, the aliphatic propyl spacer, which links ureidobenzothiazole and *N*-methylpiperazine in **AKE-5l**, was replaced by a phenyl ring to improve the anticancer spectrum while retaining the BCR-ABL inhibitory activity. The terminal phenyl was substituted with various hydrophilic and lipophilic moieties to underscore their impact on both kinase activity and cellular potency. These two major structural modifications generated a concise library of six 1–(6–(2-methoxyphenyl)benzo[*d*]thiazol-2-yl)-3-phenylurea derivatives **2a**–**f**. All target compounds were evaluated against both BCR-ABL^WT^ and BCR-ABL^T315I^, and a representative derivative **2b** was further profiled against an array of imatinib-resistant BCR-ABL mutants.

## Results and discussion

### Experimental

The detailed experimental section is included in the supplemental material.

### Chemistry

**Scheme 1.**
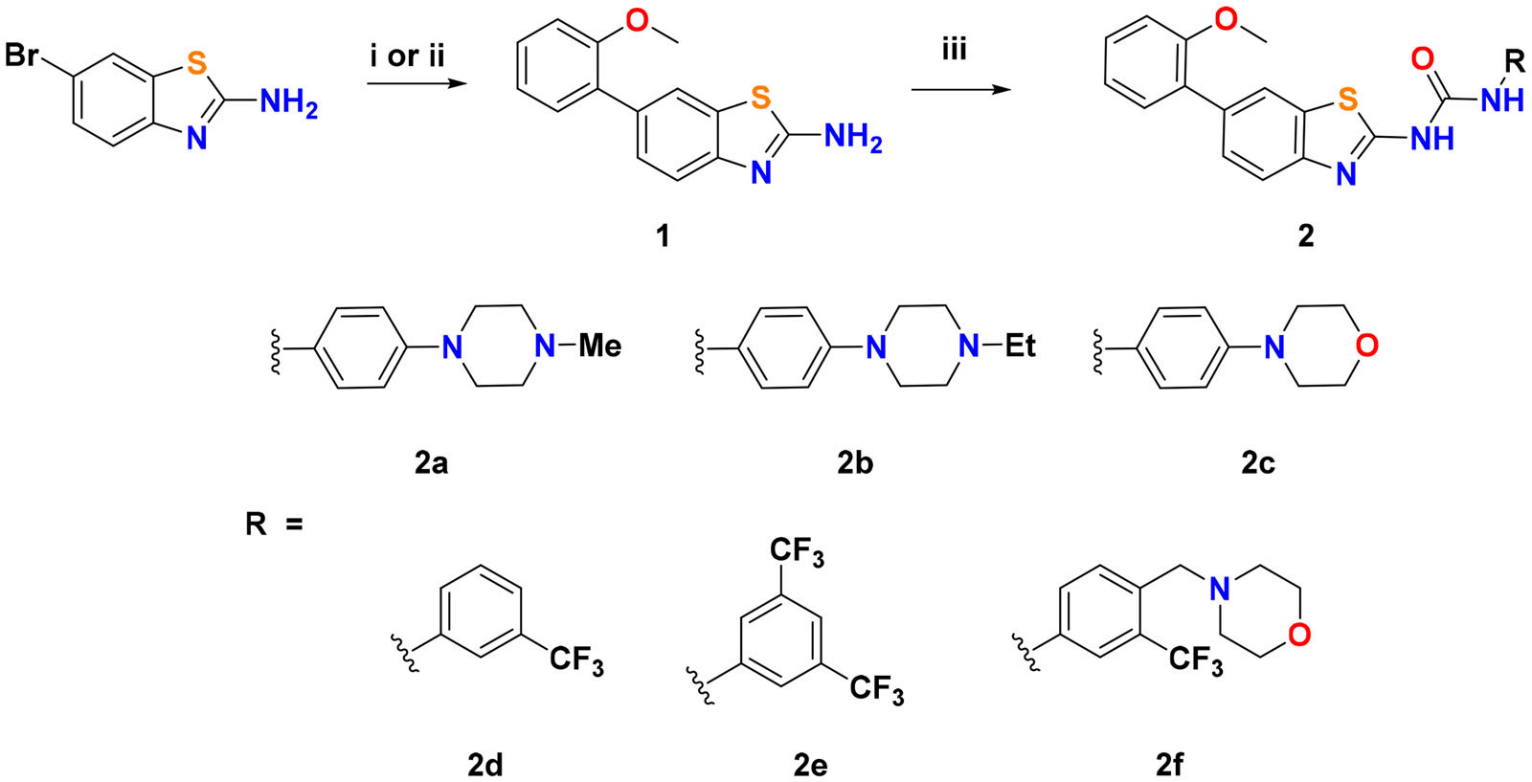
Reagents and reaction conditions: i) (2-Methoxyphenyl)boronic acid, Pd(dppf)Cl_2_• CH_2_Cl_2_, K_2_CO_3_, 1,4-dioxane:H_2_O (3:1, v:v), 95 ^0^C, 4 h, 58%; ii) (2-Methoxyphenyl)boronic acid, Pd(dppf)Cl_2_• CH_2_Cl_2_, NaHCO_3_, DME:H_2_O (3:1, v:v), 85 ^0^C, 2 h, 87% iii) a) CDI, DMF, rt, overnight; b) Appropriate substituted aniline, DMF, 100 ^0^C, 3 h, 18–76% (over two steps).

The synthetic outline for the preparation of the final compounds is illustrated in Scheme 1. The key starting material, 6-(2-methoxyphenyl)benzo[*d*]thiazol-2-amine (**1**), was obtained *via* Suzuki coupling of 2-amino-6-bromobenzothiazole with (2-methoxyphenyl)boronic acid using Pd(dppf)Cl_2_• CH_2_Cl_2_ as a catalyst under two different conditions. Initially, the reaction was conducted in 1,4-dioxane:H_2_O, (3:1, v/v) at 95 ^0^C for 4 h using potassium carbonate as a base, and afforded compound **1** in 58% yield. Changing the solvent with 1,2-dimethoxyethane (DME):H_2_O (3:1, v/v), using sodium bicarbonate and running the reaction at 85 ^0^C for 2 h furnished **1** in higher yield (87%). Treatment of benzothiazol-2-yl amine **1** with 1,1′-carbonyldiimidazole (CDI) in DMF afforded the corresponding isocyanate, which was coupled with the proper aniline at 100 ^0^C to generate the target ureidobenzothiazoles.

### *In vitro* kinase screening

All final compounds **2a**–**f** were tested against the native BCR-ABL^WT^ along with its clinically imatinib-resistant T315I mutant at Reaction Biology Corporation (RBC, Malvern, PA, USA)^26^, using imatinib as a reference compound and the pan kinase inhibitor staurosporine as a positive control (Table 1). As disclosed from the data, ureidobenzothiazoles **2a**–**c**, with hydrophilic cyclic amine at the 4-position of the terminal phenyl ring, showed excellent inhibitory potency with IC_50_ values spanning in the range of 0.701–2.16 nM and 0.651–8.11 nM against BCR-ABL^WT^ and BCR-ABL^T315I^, respectively, being superior to the lead compound **AKE-5l** (BCR-ABL^WT^ IC_50_ = 18.2 nM, BCR-ABL^T315I^ IC_50_ = 39.9 nM). Such biochemical outcomes indicate that replacing the oxypicolinamide moiety of **AKE-5l** with *o*-methoxyphenyl, along with changing the propyl spacer into phenyl, resulted in improved BCR-ABL inhibitory activity. The existence of terminal nitrogen with small alkyl substituents, **2a** and **2b**, was found to be optimal for BCR-ABL inhibition. Compound **2b**, with *N*-ethylpiperazine, exerted the best activity with sub-nanomolar IC_50_ values of 0.701 and 0.651 nM against BCR-ABL^WT^ and BCR-ABL^T315I^, respectively. It showed 2–3 folds better activity than its methylpiperazine congener **2a** (BCR-ABL^WT^ IC_50_ = 1.50 nM, BCR-ABL^T315I^ IC_50_ = 2.03 nM). Replacing the terminal nitrogen of piperazines **2a** and **2b** with oxygen **2c** retained the BCR-ABL^WT^ activity, however, it led to reduction in BCR-ABL^T315I^ inhibition (BCR-ABL^T315I^ IC_50_ = 8.11 nM). Insertion of *m*-trifluoromethyl group on the distal phenyl while removal of the hydrophilic cyclic amines **2d** resulted in a dramatic drop in potency (BCR-ABL^WT^ IC_50_ = 681 nM, BCR-ABL^T315I^ IC_50_ = 1240 nM). Such decline in activity was much more pronounced upon the introduction of an additional trifluoromethyl group at the 5-position, as in **2e** (BCR-ABL^WT^ IC_50_ = 4090 nM, BCR-ABL^T315I^ IC_50_ = 9560 nM). In contrast, incorporation of morpholine methylene at the 4-position of **2d** afforded **2f**, which restored inhibitory potency with IC_50_ values of 74.6 and 226 nM against BCR-ABL^WT^ and BCR-ABL^T315I^, respectively. These findings point out the indispensable role of cyclic amines, particularly piperazine, in achieving sound BCR-ABL inhibition for this set of ureidobenzothiazoles. This importance might be attributed to the hydrophilic nature of cyclic amines, which offers a favourable orientation towards the solvent exposure region of BCR-ABL. Such a hypothesis could be emphasised by noticing the detrimental impact of the existence of *m*-trifluoromethyl group (**2d** and **2e**) on the distal phenyl ring.

**Table 1.** *In vitro* inhibitory activity (IC_50_, nM) of the target compounds **2a**–**f**, lead **AKE-5l**, and imatinib against BCR-ABL^WT^ and BCR-ABL^T315I^ kinases.

Compound No.	IC_50_ (nM)^a^	
	BCR-ABL^WT^	BCR-ABL^T315I^
**2a**	1.50	2.03
**2b**	0.701	0.651
**2c**	2.16	8.11
**2d**	681	1240
**2e**	4090	9560
**2f**	74.6	226
**AKE-5l^b^**	18.2	39.9
**Imatinib**	176	>100000

^a^ All compounds were tested in a 10-dose IC_50_ mode with 3-fold serial dilution starting at 20 μM or 100 μM (for imatinib), and the reactions were conducted at 10 μM ATP.

^b^ Reported values.^19^

As an example of the piperazine ureidobenzothiazoles, compound **2b** was further profiled against a panel of clinically imatinib-resistant BCR-ABL mutants, including the P-loop mutants E255K and Q252H, activation loop mutant H396P, catalytic segment mutant M351T, and mutant F317I in the ATP binding region (Table 2). The obtained findings confirmed the potent ability of **2b** to inhibit the activity of the clinically relevant BCR-ABL with single-digit nanomolar IC_50_ values.

**Table 2.** *In vitro* enzymatic activity of **2b** over a panel of clinically-resistant BCR-ABL mutants.

Compound No.	IC_50_ (nM)^a^				
	ABL^E255K^	ABL^F317I^	ABL^H396P^	ABL^M351T^	ABL^Q252H^
**2b**	2.47	5.16	< 1.00	1.06	3.64

^a^ Compound **2b** was tested in a 10-dose IC_50_ mode with 3-fold serial dilution starting at 20 μM, and the reactions were carried out at 10 μM ATP.

**Figure 4.**
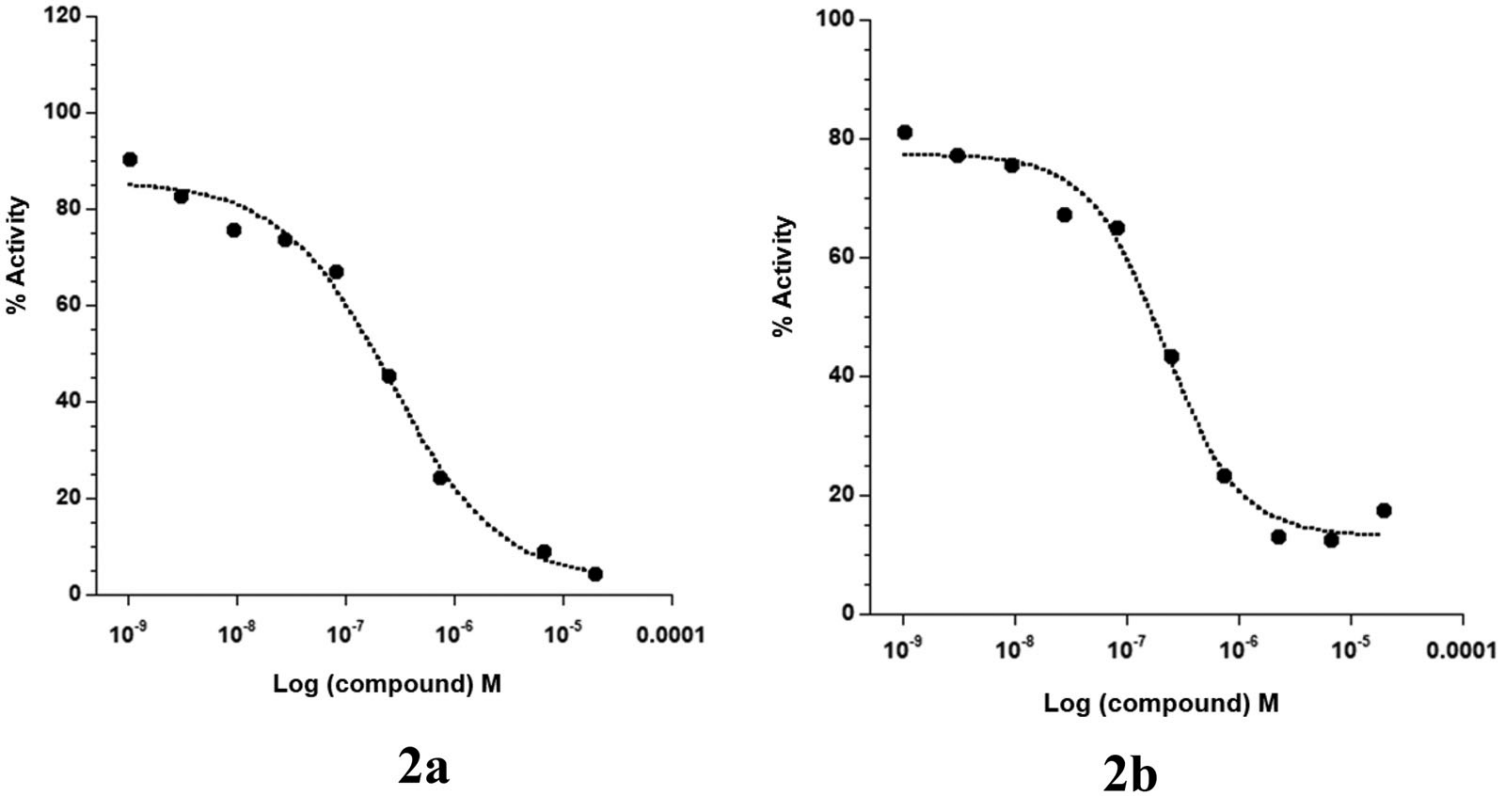
Dose-response curves of compounds **2a** and **2b** against DDR1.

On the other hand, to gain certain insights about the kinase selectivity of this array of ureidobenzothiazoles, compounds **2a** and **2b** were tested against DDR1 (Figure 4). Discoidin domain receptors (DDR1/2) are transmembrane receptor tyrosine kinases (RTKs), activated by fibrillar collagens^27^. DDR1/2 and BCR-ABL share almost 61% sequence similarities in their ATP binding domains. Therefore, several BCR-ABL inhibitors like imatinib, nilotinib, and dasatinib possess potent nanomolar DDR inhibitory effects comparable to BCR-ABL^28^. Interestingly, compounds **2a** and **2b** showed 130 and 284 folds selectivity for BCR-ABL (IC_50_ = 1.5 and 0.7 nM, respectively) over DDR1 (IC_50_ = 195 and 199 nM, respectively). This observation points out the merit of these ureidobenzothiazoles over the known BCR-ABL inhibitors in achieving exceptional selectivity towards BCR-ABL.

### In vitro cell-based evaluation of the antiproliferative activities

#### Assessment of the antiproliferative activity by MTT assay

Motivated by the promising results of cell-free assay, we examined the antiproliferative activity of target compounds against the BCR-ABL overexpressing human leukaemia K-562 cells, along with L132 normal cell by MTT (3-[4,5-dimethylthiazol-2-yl]-2,5 diphenyl tetrazolium bromide) assay (Table 3). Consistent with biochemical kinase findings, the most active ureidobenzothiazoles **2a**–**c** displayed good anticancer activity against K-562 cell with GI_50_ values of 3.39–4.52 µM. On the other hand, the *m*-trifluoromethylphenyl substituted members **2d**–**f** exerted modest cellular potency (GI_50_ > 10 µM). These cellular outcomes define the impact of BCR-ABL inhibition on the proliferation of K-562 cells. Moreover, compounds **2a**–**c** showed weak cytotoxic effects over L132 normal cell, as evidenced by high GI_50_ values (17.86–26.78 µM). In terms of selectivity index (SI), the top potent compounds **2a**–**c** had reasonable SI values spanning in the range of 5.3–6.8, which indicate their selective antiproliferative activity towards cancer cell K-562.

**Table 3.** MTT antiproliferative activity (GI_50_, μM) of compounds **2a–f** against leukaemia K-562 and L132 normal cell^a^.

Compound No.	GI_50_ (μM)		SI^c^
	K-562	L132	
**2a**	4.52	26.78	5.9
**2b**	3.39	17.86	5.3
**2c**	3.82	26.10	6.8
**2d**	>10.0	NT^b^	ND^d^
**2e**	13.88	35.05	2.5
**2f**	>10.0	NT^b^	ND^d^
**Imatinib**	0.80	>10.0	>12.5

^a^ % GI and GI_50_ were obtained after incubation for 72 h, and the presented values are the average of at least two independent measurements with standard deviations less than 20%. ^b^ NT: not tested. ^c^ SI: selectivity index (GI_50_ (L132)/GI_50_ (K562). ^d^ ND: not determined.

#### Broader assessment of anticancer activity by sulforhodamine B (SRB) assay

##### Single dose assay

**Figure 5.**
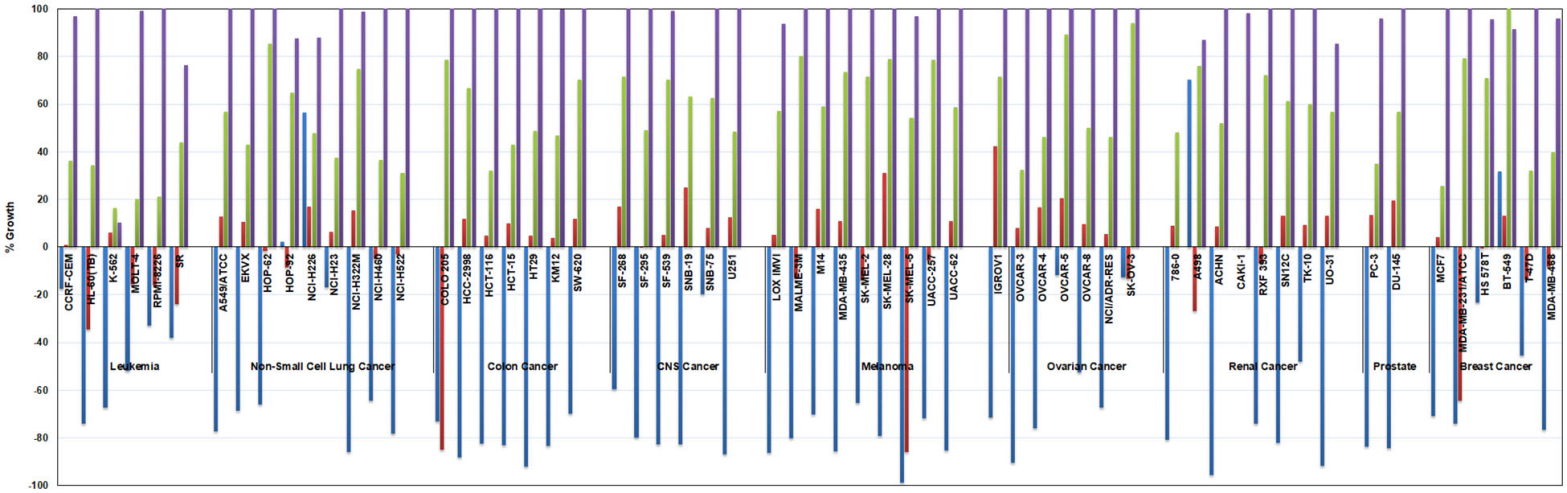
Growth percentages of the NCI-60 cell lines panel after treatment with 10 µM dose of compounds **2b** (blue), **2e** (red) **2f** (green), and lead benzothiazole **AKE-5l** (purple).

To gain further insights about the anticancer profile of this new ureidobenzothiazole chemotypes, the structures of all final compounds were submitted to the National Cancer Institute (NCI, Developmental Therapeutics Program, Bethesda, MD, USA). Based on NCI selection criteria, compounds **2b (AK-HW-90)**, **2e**, and **2f** were chosen for broad screening against a library of 60 human cancer cell lines, representing nine types of blood and solid cancers. Employing the highly sensitive sulforhodamine B (SRB) assay^29^, compounds **2b**, **2e**, and **2f** were tested initially at a single dose of 10 μM, and their detailed activities on the percentages growth of the NCI-60 cell lines were illustrated in Figure 5, and compared with that observed for the lead benzothiazole **AKE-5l**.

Interestingly, all three compounds **2b**, **2e**, and **2f** showed superior anticancer potency (low % growth values) rather the lead ureidobenzothiazole **AKE-5l**. While **AKE-5l** showed narrow spectrum activity towards only K-562 cell line (% growth = 10.22), the *o*-methoxyphenyl benzothiazoles **2b**, **2e**, and **2f** exerted broad spectrum anticancer effect against multiple cancer cells (Figure 5). Among these compounds, the most potent BCR-ABL inhibitor **2b** elicited the best cellular activity, where it manifested remarkable lethal effects (minus % growth values) over 55 cancer cells. Since both lead **AKE-5l** and **2b (AK-HW-90)** share *N*-alkylpiperazine and ureidobenzothiazoles as common structural features, it was evident that replacing oxypicolinamide moiety and propyl spacer of **AKE-5l** with *o*-methoxyphenyl and phenyl ring in **2b**, respectively, led to dramatic change in the anticancer property from narrow into expansive spectrum covering diverse cancer cells. Next in the activity rank is the 3,5-bis(trifluoromethyl)phenyl derivative **2e**, which showed cytotoxic rather cytostatic activity against 21 cancer cells. In contrast to **2b** and **2e**, compound **2f** evinced reasonable cytostatic growth inhibitory activity (% growth less than 40) towards 14 cancer cells. Despite of the modest BCR-ABL inhibitory potency of **2e** and **2f**, they showed good anticancer activity over NCI-60 cell lines, particularly **2e**, which points out the existence of other underlying mechanism(s), beside kinase inhibition, that contribute to the anticancer property of this novel chemotype.

##### Five dose assay

The promising anticancer activities of compounds **2b** and **2e** fulfilled the NCI criteria, therefore, they were advanced to five-dose testing mode to determine their GI_50_ (the molar concentration causing 50% growth inhibition), TGI (the molar concentration producing 100% GI) and LC_50_ (the molar concentration achieving 50% lethality or tumour regression). The GI_50_ values, a measure of compound’s potency, of **2b** and **2e** along with imatinib^30^ are presented in Table 4. Both compounds **2b** and **2e** showed high potency with one-digit micromolar GI_50 _values against all examined cell lines. Interestingly, **2b** and **2e** elicited superior anticancer potencies than imatinib over 55 and 54 cell lines, respectively. Regarding leukaemia, both **2b** and **2e** outperformed imatinib over five leukaemia cell lines; CCRF-CEM and MOLT-4 cells (ALL), HL-60 (TB) (AML), SR (ALK-positive anaplastic large cell lymphoma), and PRMI-8226 (multiple myeloma). For example, **2b** and **2e** showed equipotent activity against SR cell with GI_50_ value of ∼ 1.70 µM. Moreover, the growth of imatinib-insensitive lymphoma CCRF-CEM cell was potently suppressed by **2b** (GI_50_ = 1.81 µM) and **2e** (GI_50_ = 2.43 µM). The ethylpiperazine derivative **2b** exhibited significant antileukemic activity over the BCR-ABL positive cell K-562 with GI_50_ value of 0.293 µM.

**Table 4.** GI_50_ values (µM) of **2b**, **2e**, and imatinib against NCI 60-cell line panel^a,b^.

Cell lines	GI_50_ (µM)			Cell lines	GI_50_ (µM)		
	**2b**	**2e**	Imatinib		**2b**	**2e**	Imatinib
**Leukaemia**				M14	1.62	1.54	19.28
CCRF-CEM	1.81	2.43	16.98	MDA-MB-435	1.71	2.27	17.91
HL-60(TB)	1.73	2.01	13.49	SK-MEL-2	1.94	2.09	25.00
K-562	0.293	1.54	0.02	SK-MEL-28	1.76	2.51	14.62
MOLT-4	1.80	1.59	5.13	SK-MEL-5	1.73	1.57	12.13
RPMI-8226	2.06	1.63	6.05	UACC-257	2.11	2.31	21.13
SR	1.65	1.76	7.14	UACC-62	1.87	1.56	19.01
**Non-Small Cell Lung Cancer**				**Ovarian Cancer**			
A549/ATCC	1.98	3.19	24.49	IGROV1	1.87	2.83	21.18
EKVX	1.51	NT	26.18	OVCAR-3	1.64	1.58	34.20
HOP-62	NT	2.10	21.53	OVCAR-4	1.68	2.12	20.04
HOP-92	1.47	1.19	13.34	OVCAR-5	1.80	2.54	0.58
NCI-H226	2.09	2.16	18.11	OVCAR-8	1.99	3.04	27.67
NCI-H23	1.67	2.02	14.96	NCI/ADR-RES	1.72	2.37	22.96
NCI-H322M	1.53	2.78	22.80	SK-OV-3	1.79	2.92	28.91
NCI-H460	1.77	2.26	16.18	**Renal Cancer**			
NCI-H522	1.75	1.76	16.41	786-0	1.80	1.44	16.00
**Colon Cancer**				A498	1.80	1.64	21.98
COLO 205	1.97	1.65	17.62	ACHN	1.74	2.14	25.23
HCC-2998	1.83	2.65	21.23	CAKI-1	NT	2.33	33.96
HCT-116	1.58	1.07	12.59	RXF 393	1.70	2.19	15.07
HCT-15	1.20	2.55	19.95	SN12C	1.77	1.70	33.19
HT29	1.31	2.15	3.97	TK-10	2.12	4.03	26.85
KM12	1.75	2.07	18.84	UO-31	1.52	1.79	22.34
SW-620	1.94	3.04	23.44	**Prostate Cancer**			
**CNS Cancer**				PC-3	1.79	1.49	21.38
SF-268	1.87	2.86	26.30	DU-145	1.74	4.00	18.79
SF-295	1.68	2.64	19.77	**Breast Cancer**			
SF-539	1.60	1.99	10.57	MCF7	1.31	2.28	18.24
SNB-19	2.03	2.44	38.55	MDA-MB-231	1.72	1.95	18.66
SNB-75	1.49	2.25	20.28	HS 578T	1.95	2.35	14.59
U251	1.57	2.31	17.99	BT-549	1.88	1.62	16.11
**Melanoma**				T-47D	1.58	2.16	19.91
LOX IMVI	1.70	1.68	18.11	MDA-MB-468	2.17	1.52	NT^b^
MALME-3M	1.71	1.81	16.33				

^a^ Data were obtained from the National Cancer Institute (NCI) *in vitro* disease-oriented human tumour cell line screen (five-dose–response curve) for compounds **2b** (NSC code: 795187), **2e** (NSC code: 795906), and imatinib mesylate (NSC code: 743414).

^b^ NT: not tested.

From another perspective, it was documented that imatinib could ameliorate the hypoxic circumstances in non-small cell lung cancer (NSCLC)^31^, beside its ability to improve drug delivery and efficacy in NSCLC xenografts^32^. In this aspect, it is interesting to observe that both compounds **2b** and **2e** possess superior potency to imatinib over seven of the tested NSCL cancer cells. For instance, compounds **2b** and **2e** displayed GI_50_ values of 1.47 µM and 1.19 µM against HOP-92 cell line, respectively, with 9–11 folds better potency than imatinib (GI_50_ = 13.34 µM). The ethylpiperazine member **2b** slightly surpassed the potency of its 3,5-bis-trifluoromethyl congener **2e** over six cell lines of NSCL cancer panel. Compound **2b** exerted equipotent anticancer effects (GI_50_ ∼ 1.76 µM) over NCI-H460 and NCI-H522 cell lines.

On the other hand, imatinib has shown beneficial clinical effects for the treatment of certain metastatic/inoperable gastrointestinal stromal tumours (GIST)^33^. In this aspect, it is noteworthy reporting that both **2b** and **2e** elicited considerable higher anticancer activities than imatinib over all tested colon cancer cell lines. The growth of HT29, the most imatinib-responsive colorectal cancer cell, was strongly inhibited by **2b** and **2e**, with GI_50_ values of 1.31 µM and 2.15 µM, respectively. While imatinib exerted modest anticancer activity over COLO205, HCC-2998, HCT-116 and multidrug-resistant HCT-15 colon cancer cells (GI_50_ = 12.59–21.23 µM), **2b** and **2e** showed single-digit micromolar GI_50_ values spanning in the range of 1.07–2.65 µM. Such promising cellular findings recommend further investigations of **2b** and **2e** as potential candidates for colorectal cancer.

Since glioblastoma (GBM), the most aggressive form of brain tumours^34^, is resistant to the majority of tyrosine kinase inhibitors, including imatinib^35^, it is important to point out that both **2b** and **2e** were remarkably superior to imatinib across all six examined brain cancer cell lines. Moreover, **2b** and **2e** exerted good anticancer effects against the temozolomide (TMZ)-resistant cell lines SF-295, SNB-75, SNB-19, and SF-268 gliomas. For instance, the ethyl piperazine member **2b** suppressed the growth of the SNB-19 cell line with a GI_50_ value of 2.03 µM. Referring to melanoma, both **2b** and **2e** showed comparable potencies against all tested melanoma cell lines, surpassing imatinib, with GI_50_ values of 1.54–2.51 µM.

Moreover, imatinib efficacy in the treatment of ovarian cancers as a combination with docetaxel was reported^36^. In this line, it is interesting to find that compounds **2b** and **2e** have significant anticancer activity outperforming imatinib over all tested ovarian cancer cells, except OVCAR-5. Particularly, **2b** showed GI_50_ values less than 2.0 µM over all cell lines. Of special interest, ureidobenzothiazoles **2b** and **2e** exerted appreciable potency towards the paclitaxel-resistant NCI/ADR-RES cell line, with GI_50_ values of 1.72 µM and 2.37 µM, respectively.

Beside the aforesaid-targeted cellular potencies of **2b** and **2e**, they elicited broad-spectrum activities over multiple cell lines originating from different cancer tissues. As an example, multidrug-resistant (MDR) UO-31 and RXF-393 renal cells showed high sensitivity to **2b** (GI_50_ = 1.52 µM and 1.70 µM) and **2e** (GI_50_ = 1.79 µM and 2.19 µM), respectively. Moreover, the top active member **2b** exhibited pronounced anticancer potencies over the prostate cancer cell PC-3, MCF7, and triple-negative breast cancer cell MDA-MB-468 with GI_50_ values of 1.79, 1.31, and 2.17 µM, respectively.

Regarding the efficacy parameters (TGI and LC_50_ values) of ureidobenzothiazoles **2b** and **2e** (Table 5), it was found that compound **2b** with hydrophilic ethylpiperazine motif possesses better efficacy than **2e** against several cancer cells with TGI < 5.0 µM and LC_50_ < 10.0 µM. For example, **2b** induced total growth inhibition against K-562 and HCT-15 cell lines, with TGI values of 1.34 and 2.47 µM, respectively.

**Table 5.** TGI and LC_50_ values (µM) of **2b** and **2e** over the most sensitive cell lines^a,b^. 2b

Cancer Panel/Cell line			2e	
	TGI	LC_50_	TGI	LC_50_
***Leukaemia***				
K-562	**1.34**	ND	8.95	43.9
MOLT-4	**3.67**	7.45	7.24	51.3
**NSCL Cancer**				
NCI-H322M	**3.03**	5.99	9.06	37.0
NCI-H460	**3.27**	6.01	6.12	23.5
***Colon Cancer***				
HCT-15	**2.47**	5.06	10.6	34.6
HT29	**2.70**	5.57	9.21	33.0
***CNS Cancer***				
SNB-75	**2.86**	5.46	11.8	45.3
U251	**2.94**	5.51	10.3	34.4
***Melanoma***				
M14	**3.03**	5.66	**3.22**	6.73
SK-MEL-5	**3.12**	5.63	**2.98**	5.64
***Ovarian Cancer***				
OVCAR-3	**3.11**	5.90	**4.58**	16.7
OVCAR-5	**3.37**	6.33	9.66	32.7
***Renal Cancer***				
ACHN	**3.11**	5.58	7.71	28.2
UO-31	**2.92**	5.63	**4.07**	9.29
***Prostate Cancer***				
DU-145	**3.26**	6.08	14.9	42.4
***Breast Cancer***				
MCF7	**2.86**	6.25	10.9	46.2

^a^ Data were obtained from the National Cancer Institute (NCI), based on the five-dose–response curve of compounds **2b** and **2e**, bold figures refer to TGI values less than 5 µM, and underlined figures indicate LC_50_ less than 10 µM.

^b^ NT: not tested.

### Molecular docking study

**Figure 6.**
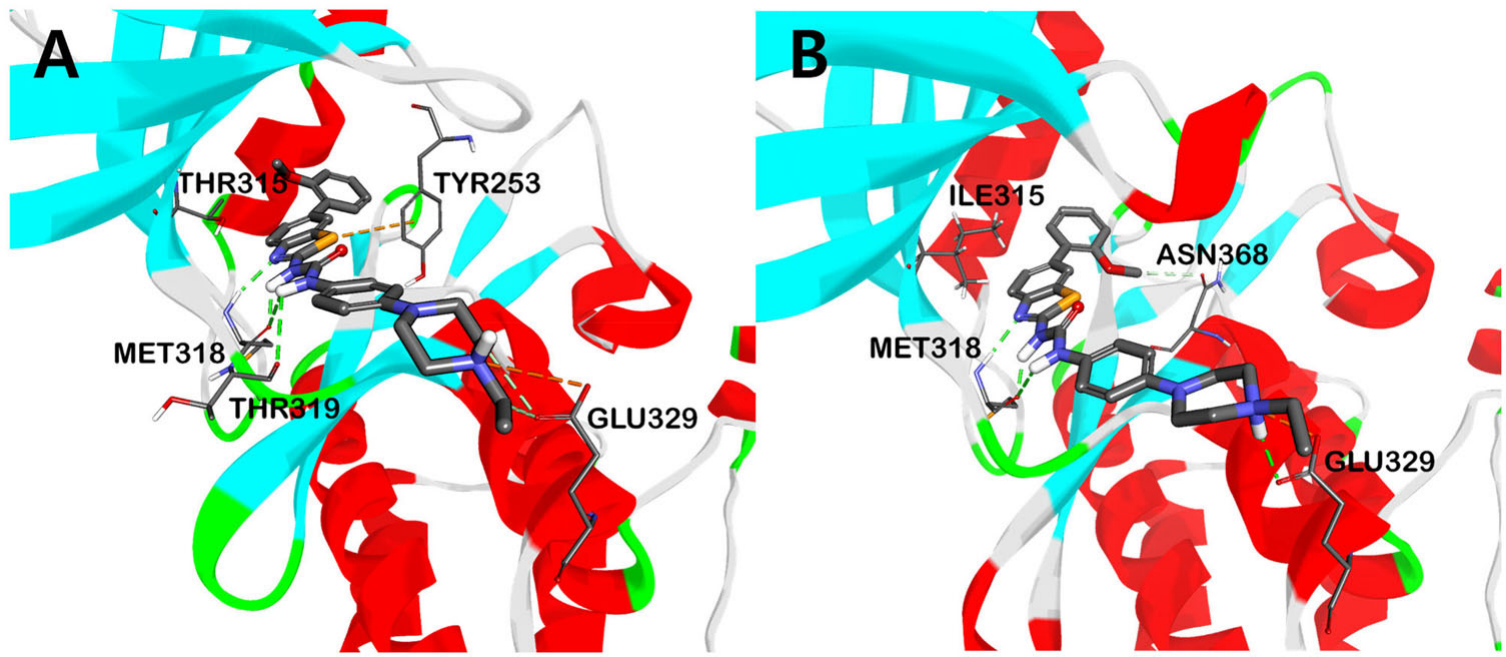
The putative binding mode of compound **2b** in the kinase domain of BCR-ABL^WT^ (**A**) and BCR-ABL^T315I^ (**B**). The hydrogen bond interactions are shown in green dotted lines.

To get insights about the binding mode of this new set of ureidobenzothiazoles, molecular docking for the most potent candidate **2b** (**AK-HW-90**) into the catalytic kinase domain of wild-type BCR-ABL (PDB code: 2GQG)^37^ and its T315I mutant (PDB code: 2Z60)^38^, in their DFG-in conformation, was conducted. As shown in Figure 6, compound **2b** showed tight binding within the ATP binding site of both BCR-ABL^WT^ and BCR-ABL^T315I^ kinases, as demonstrated by the formation of crucial three hydrogen bonds (HB) with hinge region residue Met318. The ring nitrogen of the benzothiazole scaffold was engaged in HB with amide hydrogen of Met318, while the two NH hydrogens of urea moiety formed two HBs with the amino carbonyl oxygen of Met318. In addition, attractive charge interaction was noticed between the terminal nitrogen of ethyl piperazine and the side chain carboxyl group of Glu329 at the solvent-accessible channel of the BCR-ABL kinase. Moreover, the methoxy group shared carbon-hydrogen bonds with the side chain hydroxyl group of Thr315 in BCR-ABL^WT^. However, in the case of BCR-ABL^T315I^, due to steric hindrance with Ile315, which has a larger residual volume than Thr315, such carbon-hydrogen interactions were lacking. Instead, π-alkyl interactions were observed between the methoxy group and the side chain phenyl group of the Tyr253 residue (Figure S1). Besides, in the case of BCR-ABL^WT^, π-sulphur interaction was noticed between the sulphur atom of benzothiazole and the phenyl group of the Tyr253 residue. The aforementioned binding interactions might justify the sub-nanomolar potency of **2b** against both BCR-ABL^WT^ and its T315 mutant.

### In silico bioavailability prediction

The bioavailability of compound **2b** was predicted by SwissADME online platform^39^. The bioavailability radar panel evaluates the bioactive candidates in terms of their physicochemical parameters, such as lipophilicity, flexibility, polarity insolubility, size, and saturation. Interestingly, compound **2b** was found to be in the pink region (optimal ranges) with favourable drug-like properties (Figure S2).

## Conclusion

In the current investigation, we report the design and synthesis of new phenyl-ureidobenzothiazoles **2a**–**f** in an effort to improve the BCR-ABL inhibitory activity of our previously reported lead **AKE-5l**. Two major structural modifications of **AKE-5l** were undertaken herein, replacing the 6-oxypicolinamide moiety and the propyl spacer of **AKE-5l** with *o*-methoxyphenyl and phenyl ring, respectively. Among the target compounds, both **2a** and its ethylpiperazine congener **AK-HW-90** (**2b**) stood as the most potent members with IC_50_ values of 2.0 and 0.65 nM against BCR-ABL^T315I^, respectively, surpassing the activity of lead **AKE-5l** (IC_50_ = 39.9 nM). Furthermore, **AK-HW-90** (**2b**) potently inhibited the activity of a panel of imatinib-resistant mutants with single digit nanomolar IC_50_ values. On the cellular level, in contrast to the narrow spectrum anticancer activity of the lead **AKE-5l**, compound **AK-HW-90** (**2b**) exerted considerable broad-spectrum anticancer activity across multiple cell lines including the CML K-562 cell. In addition, **AK-HW-90** (**2b**) surpassed the activity of imatinib over more than 50 cancer cell lines with single digit micromolar GI_50_ values. Molecular docking study of **AK-HW-90** (**2b**) was conducted to get insights about its postulated binding mode with BCR-ABL^WT^ and BCR-ABL^T315I^. Considering the cell-free and cell-based assay results, compound **AK-HW-90** (**2b**) may serve as a promising chemotherapeutic candidate for CML and other cancer types.

## Supplementary Material

Supplemental Material
