# Supplementary material for: Overcoming the imatinib-resistant BCR-ABL mutants with new ureidobenzothiazole chemotypes endowed with potent and broad-spectrum anticancer activity
Source: J Enzyme Inhib Med Chem. 2023 Mar 17;38(1):2189097. doi: 10.1080/14756366.2023.2189097 (PMC10026764; doi:10.1080/14756366.2023.2189097)
Supplement: Supplemental Material [file IENZ_A_2189097_SM3161.pdf]

# Supporting Information

# Overcoming the imatinib-resistant BCR-ABL mutants with new ureidobenzothiazole chemotypes endowed with potent and broad-spectrum anticancer activity

Ashraf K. El-Damasy [a,b 1]*, Heewon Jin [a, 1], Jung Woo Park[c], Hyun Ji Kim [a], Hanan Khojah[d], Seon Hee Seo [e], Ju-Hyeon Lee[a], Eun-Kyoung Bang [a], Gyochang Keum [a,f]*

[a] *Center for Brain Technology, Brain Science Institute, Korea Institute of Science and Technology (KIST), Seoul 02792, Republic of Korea*
[b] *Department of Medicinal Chemistry, Faculty of Pharmacy, Mansoura University, Mansoura 35516, Egypt*
[c] *Center for Supercomputing Applications, Div. of National Supercomputing R&D, Korea Institute of Science and Technology Information, 245, Daehak-ro, Yuseong-gu, Daejeon, 34141, Republic of Korea*
[d] *Department of Pharmacognosy, College of Pharmacy, Jouf University, P.O. Box 2014, Sakaka, Al-Jouf, Saudi Arabia*
[e] *Center for Brain Disorders, Brain Science Institute, Korea Institute of Science and Technology (KIST), Seoul 02792, Republic of Korea*
[f] *Division of Bio-Medical Science & Technology, KIST School, Korea University of Science and Technology (UST), Seoul 02792, Republic of Korea*

[1] These authors contributed equally to this work.

**Corresponding author:**
Ashraf K. El-Damasy, E-mail: ph_karem2000@mans.edu.eg, phkarem2006@gmail.com
Gyochang Keum, E-mail: gkeum@kist.re.kr

**Contents** **Pages**

| Contents | Pages |
| --- | --- |
| 1) Experimental section | 2–3 |
| 2) $^{1}$H NMR and $^{13}$C NMR spectra | 4–10 |
| 3) HRMS charts | 11–13 |
| 4) 2D binding mode & bioavailability radar of **2b** | 14 |
| 5) NCI data | 15–19 |
| 6) References | 20 |

## 1. Experimental section

*1.1. General*

All reactions and manipulations were conducted utilizing standard Schlenk techniques. All solvents and reagents were obtained from commercial suppliers and were used without further purification. The reaction progress was monitored on TLC plate (Merck, silica gel 60 $F_{254}$). Flash column chromatography was carried out using silica gel (Merck, 230–400 mesh) and the eluent solvents are indicated as a mixed solvent with either given percentage or volume-to-volume ratios. $^{1}H$ and $^{13}C$ NMR spectra were recorded on a Bruker Avance 400 MHz spectrometer, using the proper deuterated solvents, as noted. Chemical shifts (δ) are given in parts per million (ppm) upfield from tetramethylsilane (TMS) as internal standard. s, d, t, and m refer to singlet, doublet, triplet and multiplet, respectively, and coupling constants (*J*) are reported in hertz (Hz). High resolution mass spectra (HRMS) were recorded on JMS 700 (Jeol, Japan) mass spectrometer, with magnetic sector-electric sector double focusing mass analyzer, and FAB+ ion mode. The purity of all final compounds was found to be > 95%, as determined by $^{1}H$ NMR. 3-(Trifluoromethyl)aniline and 3,5-bis(trifluoromethyl)aniline were commercially available, while the cyclic amine bearing anilines were synthesized adopting the reported procedure \.

*1.2. 6-(2-Methoxyphenyl)benzo[d]thiazol-2-amine* (**1**)
A solution of 2-methoxyphenyl boronic acid (199 mg, 1.31 mmol) in dimethoxyethane:$H_2O$ (3:1, v/v, 3 mL) was added to a stirred solution of 6-bromo-2-aminothiazol (200 mg, 0.873 mmol), $NaHCO_3$ (220 mg, 2.619 mmol), and Pd(dppf)$Cl_2$·$CH_2Cl_2$ (143 mg, 0.20 mmol) in dimethoxyethane:$H_2O$ (3:1, v/v, 1 mL). The reaction mixture was stirred at room temperature for 85 min at 80–85 ºC. After the reaction completion, the solvent was evaporated under vacuum, and the residue was filtered by celite pad. The filtrate was evaporated and the resulting residue was purified by column chromatography using (33–50% ethyl acetate in hexane) to afford the title compound as a brown solid; 194 mg (86.6%); $^{1}H$ NMR (400 MHz, $CDCl_3$) δ 7.77 (d, *J* = 1.4 Hz, 1H), 7.58 (d, *J* = 8.3 Hz, 1H), 7.48 (dd, *J* = 8.3, 1.6 Hz, 1H), 7.35–7.30 (m, 2H), 7.04 (td, *J* = 7.5, 0.84 Hz, 1H), 7.00 (d, *J* = 8.2 Hz, 1H), 5.42 (br. s, 1H), 3.83 (s, 3H); $^{13}C$ NMR (100 MHz, $CDCl_3$) δ 167.0, 156.4, 146.7, 133.8, 130.9, 129.9, 129.2, 128.8, 128.3, 122.2, 120.9, 117.4, 111.3, 55.6.

*1.3. General procedure for synthesis of compounds **2a–f***
A solution of compound **1** (200 mg, 0.78 mmol) and 1,1'-carbonyldiimidazole (253 mg, 1.56 mmol) in anhydrous dimethylformamide (2 mL) was stirred at room temperature for 15 h. Then, the appropriate aniline (1.56 mmol) was added and the reaction mixture was heated to 100 ºC for 2.5 h. After cooling to room temperature, the reaction was quenched with water (20 mL). The aqueous layer was extracted with ethyl acetate (3×30 mL), and the organic layers were combined, washed with water and brine, dried over anhydrous $Na_2SO_4$, and filtered. The solvent was evaporated under vacuum, and the resultant residue was purified by flash column chromatography using the proper eluent to afford the target compounds in pure form.

*1.3.1. 1-(6-(2-Methoxyphenyl)benzo[d]thiazol-2-yl)-3-(4-(4-methylpiperazin-1-yl)phenyl)urea* (**2a**)
Column chromatography was performed using 5% methanol in DCM. White solid (28.1% yield); $^{1}H$ NMR (400 MHz, $CDCl_3$) δ 7.88 (s, 1H), 7.73 (d, *J* = 8.4 Hz, 1H), 7.54 (d, *J* = 7.6 Hz, 1H), 7.36–7.31 (m, 4H), 7.05–6.99 (m, 2H), 6.86 (d, *J* = 8.4 Hz, 2H), 3.82 (s, 3H), 3.17 (t, *J* = 5.0 Hz, 4H), 2.61 (t, *J* = 4.6 Hz, 4H), 2.38 (s, 3H); $^{13}C$ NMR (100 MHz, $CDCl_3$) δ 161.4, 156.5, 152.8, 148.3, 147.8, 134.1, 131.0, 130.9, 130.0, 129.8, 128.7, 128.1, 122.2, 122.1,121.0, 119.3, 116.8, 111.3, 55.6, 55.0, 49.3, 46.0; HRMS (FAB) m/z calcd for $C_{26}H_{28}N_5O_2S$ $[M+H]^+$: 474.1963, found: 474.1960.

*1.3.2. 1-(4-(4-Ethylpiperazin-1-yl)phenyl)-3-(6-(2-methoxyphenyl)benzo[d]thiazol-2-yl)urea* (**2b**)
Column chromatography was performed using 3–7% methanol in dichloromethane (DCM). White solid (23.1% yield); $^{1}H$ NMR (400 MHz, $CDCl_3$) δ 10.77 (br. s, 1H), 7.87 (s, 1H), 7.71 (d, *J* = 8.0 Hz, 1H), 7.52 (d, *J* = 7.6 Hz, 1H), 7.32–7.26 (m, 4H), 7.04–6.97 (m, 2H), 6.83 (d, *J* = 7.6 Hz, 2H), 3.81 (s, 3H), 3.14 (s, 4H), 2.59 (s, 4H), 2.48 (q, *J* = 6.9 Hz, 2H), 1.13 (t, *J* = 6.4 Hz, 3H); $^{13}C$ NMR (100 MHz, $CDCl_3$) δ 161.5, 156.5, 152.8, 148.4, 147.7, 134.1, 131.1, 130.0, 129.8, 128.7, 128.1, 122.1, 122.0, 121.9, 121.0, 119.2, 116.7, 111.3, 55.6, 52.8, 52.4, 49.5, 12.0; HRMS (FAB) m/z calcd for $C_{27}H_{30}N_5O_2S$ $[M+H]^+$: 488.2120, found: 488.2117.

*1.3.3. 1-(6-(2-Methoxyphenyl)benzo[d]thiazol-2-yl)-3-(4-morpholinophenyl)urea* (**2c**)
Column chromatography was performed using 3–7% methanol in DCM. White solid (23% yield); $^{1}H$ NMR (400 MHz, DMSO-$d_6$) δ 10.85 (br. s, 1H), 8.96 (s, 1H), 7.98 (s, 1H), 7.67 (d, *J* = 8.4 Hz, 1H), 7.50 (d, *J* = 7.6 Hz, 1H), 7.39–7.34 (m, 4H), 7.14 (d, *J* = 8.4 Hz, 1H), 7.05 (t, *J* = 7.2 Hz, 1H), 6.93 (d, *J* = 8.8 Hz, 2H), 3.79 (s, 3H), 3.36 (s, 4H), 3.09 (s, 4H); $^{13}C$ NMR (100 MHz, DMSO-$d_6$) δ 160.4, 156.6, 152.6, 147.8, 133.4, 131.6, 131.1, 130.6, 130.1, 129.2, 128.0, 122.4, 121.3, 120.8, 119.1, 116.5, 116.4, 112.2, 60.8, 59.0, 56.0.

*1.3.4. 1-(6-(2-Methoxyphenyl)benzo[d]thiazol-2-yl)-3-(3-(trifluoromethyl)phenyl)urea* (**2d**)

Column chromatography was performed using ethyl acetate-hexane (1:3, v/v). White solid (43% yield); $^1$H NMR (400 MHz, DMSO-$d_6$) δ 11.27 (br. s, 1H), 9.59 (s, 1H), 8.09 (s, 1H), 7.99 (s, 1H), 7.76 (d, $J$ = 8.4 Hz, 1H), 7.66 (d, $J$ = 8.4 Hz, 1H), 7.58 (t, $J$ = 8.0 Hz, 1H), 7.52 (dd, $J$ = 8.4, 1.2 Hz, 1H), 7.41 (d, $J$ = 7.6 Hz, 1H), 7.36 (s, 1H), 7.34 (s, 1H), 7.13 (d, $J$ = 8.4 Hz, 1H), 7.05 (t, $J$ = 7.4 Hz, 1H), 3.79 (s, 3H); $^{13}$C NMR (100 MHz, DMSO-$d_6$) δ 156.6, 140.2, 133.7, 132.5, 131.0, 130.5, 130.3, 130.0, 129.9, 129.2, 128.2, 126.0, 123.3, 123.0, 122.6, 121.3, 119.6, 119.5, 115.3, 115.3, 112.2, 56.0; HRMS (FAB) m/z calcd for $C_{22}H_{17}F_3N_3O_2S$ $[M+H]^+$: 444.0993, found: 444.0992.

*1.3.5. 1-(3,5-Bis(trifluoromethyl)phenyl)-3-(6-(2-methoxyphenyl)benzo[d]thiazol-2-yl)urea* (**2e**)
Column chromatography was performed using 1% methanol in DCM. White solid (76% yield); $^1$H NMR (400 MHz, DMSO-$d_6$) δ 11.89 (br. s, 1H), 9.96 (s, 1H), 8.31 (s, 2H), 7.96 (s, 1H), 7.71 (s, 1H), 7.62 (d, $J$ = 8.0 Hz, 1H), 7.52 (dd, $J$ = 8.4, 1.2 Hz, 1H), 7.35 (d, $J$ = 8.0 Hz, 2H), 7.14 (d, $J$ = 8.0 Hz, 1H), 7.05 (t, $J$ = 7.4 Hz, 1H), 3.79 (s, 3H); $^{13}$C NMR (100 MHz, DMSO-$d_6$) δ 156.6, 141.9, 133.8, 131.7, 131.4, 131.1, 131.0, 130.7, 129.9, 129.3, 128.4, 125.1, 122.8, 122.4, 121.3, 119.0, 115.6, 112.2, 56.0; HRMS (FAB) m/z calcd for $C_{23}H_{16}F_6N_3O_2S$ $[M+H]^+$: 512.0867, found: 512.0866.

*1.3.6. 1-(6-(2-Methoxyphenyl)benzo[d]thiazol-2-yl)-3-(4-(morpholinomethyl)-3-(trifluoromethyl)phenyl)urea* (**2f**)
Column chromatography was performed using ethyl acetate-hexane (1:1, v/v). Yellow solid (18.4% yield); $^1$H NMR (400 MHz, $CDCl_3$) δ 7.94 (d, $J$ = 1.6 Hz, 1H), 7.91 (br. s, 1H), 7.84–7.73 (m, 3H), 7.63 (dd, $J$ = 8.4, 1.6 Hz, 1H), 7.40–7.36 (m, 2H), 7.09 (d, $J$ = 7.2 Hz, 1H), 7.04 (d, $J$ = 8.8 Hz, 1H), 3.87 (s, 3H), 3.76 (t, $J$ = 4.4 Hz, 4H), 3.66 (s, 2H), 2.52 (t, $J$ = 4.4 Hz, 4H); $^{13}$C NMR (100 MHz, $CDCl_3$) δ 161.4, 156.5, 152.8, 148.4, 147.8, 134.3, 134.1, 131.0, 131.0, 130.1, 129.7, 128.8, 128.7, 128.2, 128.1, 122.3, 122.1, 121.0, 119.3, 116.8, 111.3, 55.6, 55.0, 49.3, 46.0; HRMS (FAB) m/z calcd for $C_{27}H_{26}F_3N_4O_3S$ $[M+H]^+$: 543.1677, found: 543.1681.

## *1.4. In vitro biological evaluations*

### *1.4.1. In vitro kinase screening*

Reaction Biology Corporation (RBC) Kinase HotSpot$^{SM}$ service was employed for biochemical kinase evaluation of the target compounds following the reported assay protocol [5].

### *1.4.2. In vitro cell based assays*

#### *1.4.2.1. Preliminary MTT evaluation of antiproliferative activity*

The antiproliferative activity of the target benzothiazoles was assessed against human leukemia K562 cancer cell as well as L132 normal cell line, using the MTT assay adopting the reported assay protocols [6].

#### *1.4.2.2. Anti-cancer screening at NCI*

The anticancer screening of certain selected target compounds over a full panel of 60-human cancer cell lines was conducted at the National Cancer Institute (NCI), Bethesda, Maryland, USA using Sulforhodamine B (SRB) assay adopting the standard protocol [7].

## *1.5. In silico studies*

### *1.5.1. Molecular docking*

The docking models of compound **2b** were constructed utilizing the X-ray crystal structure of BCR-ABL$^{WT}$ (PDB: 2GQG) [8] or BCR-ABL$^{T315I}$ (PDB: 2Z60) [9], in its DFG-in conformation using Discovery Studio 2022 (DS). The protein structure of BCR-ABL$^{WT}$ and BCR-ABL$^{T315I}$ were prepared for docking by employing protocol "prepare protein", and ligands were prepared through protonation at pH 7.4 and energy minimization. The binding site was defined based on the ligand interactions with BCR-ABL kinase domain. The ligands were docked into the defined binding sites using the CDOCKER algorithm, and the ligand pose with the best score was selected for analysis of the binding mode.

### *1.5.2. Bioavailiability Predicition*

The bioavailability prediction of compound **2b** was assessed at http://www.swissadme.ch/index.php (accessed on 10 December 2022).

2) $^{1}H$ NMR and $^{13}C$ NMR spectra

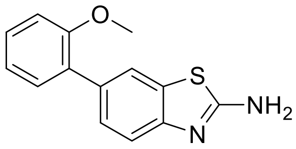


1

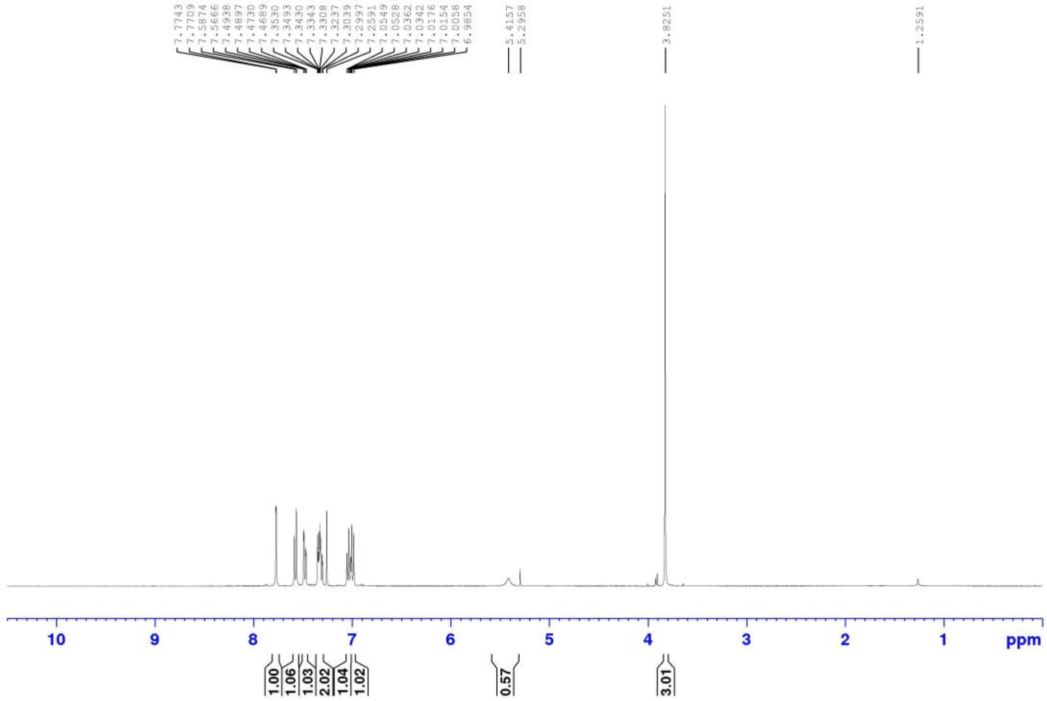


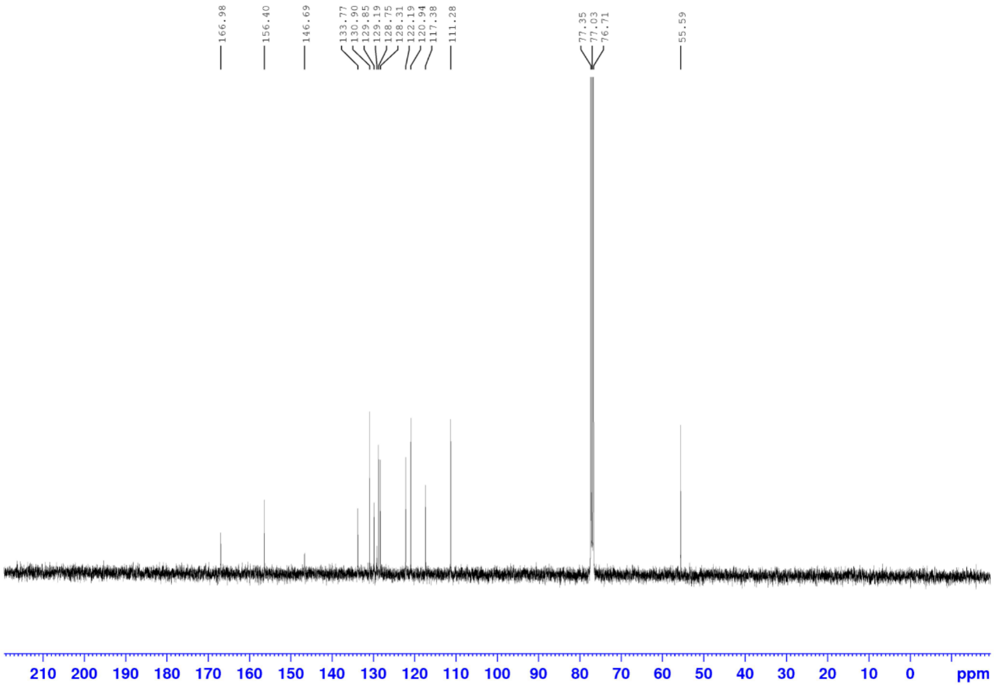

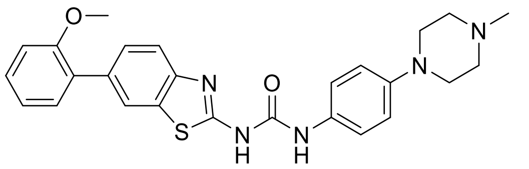

2a

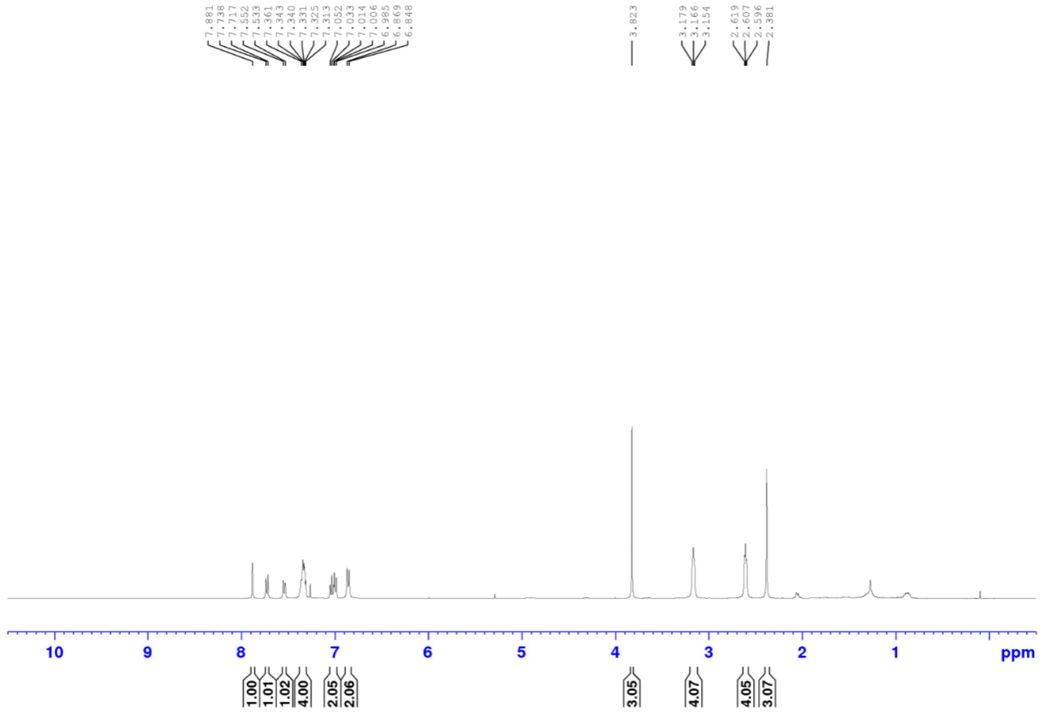
7.881
7.738
7.717
7.552
7.533
7.361
7.343
7.340
7.331
7.325
7.313
7.052
7.033
7.014
7.006
6.985
6.869
6.848
3.823
3.179
3.166
3.154
2.619
2.607
2.596
2.381
10 9 8 7 6 5 4 3 2 1 ppm
1.00
1.01
1.02
4.00
2.05
2.06
3.05
4.07
4.05
3.07

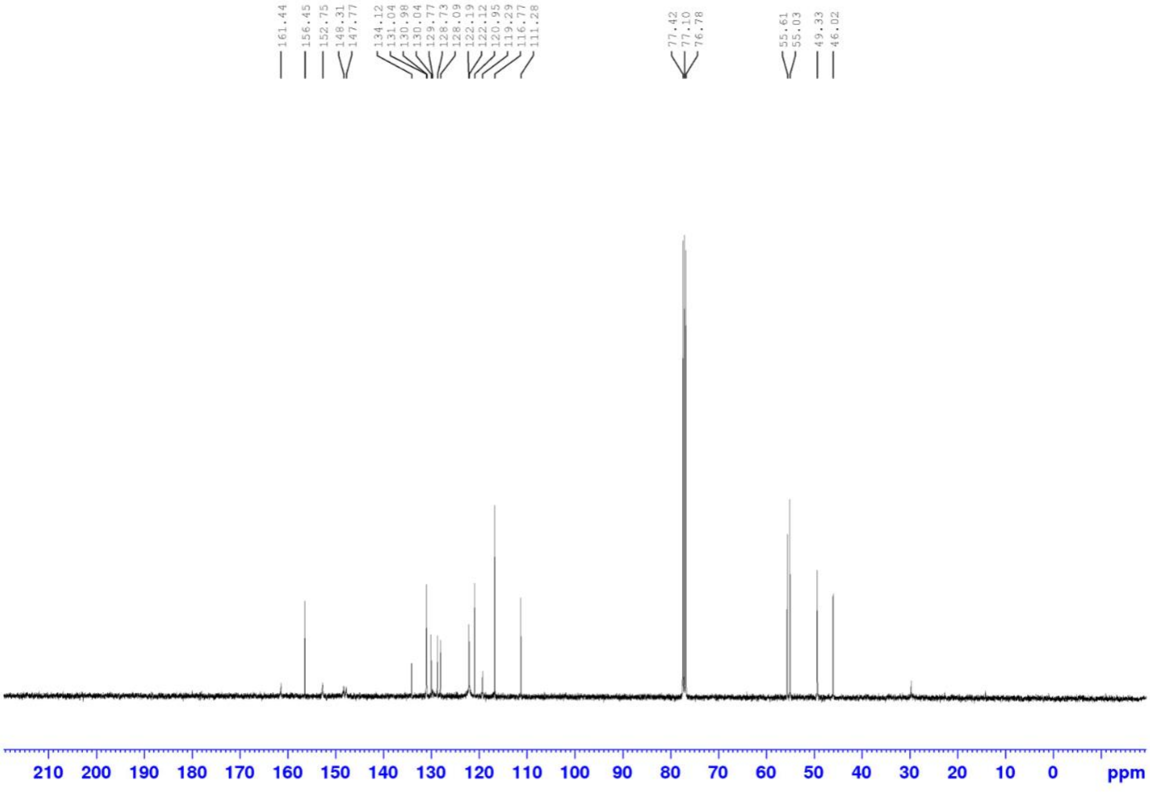
161.44
156.45
152.75
148.31
147.77
134.12
131.04
130.98
130.04
129.77
128.73
128.09
122.19
122.12
120.95
119.29
116.77
111.28
77.42
77.10
76.78
55.61
55.03
49.33
46.02
210 200 190 180 170 160 150 140 130 120 110 100 90 80 70 60 50 40 30 20 10 0 ppm

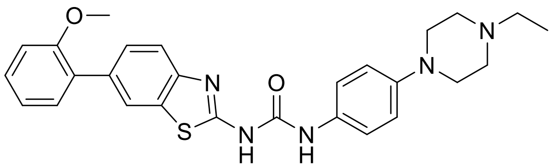

O
N
S
N
H
O
N
H
N
N


2b

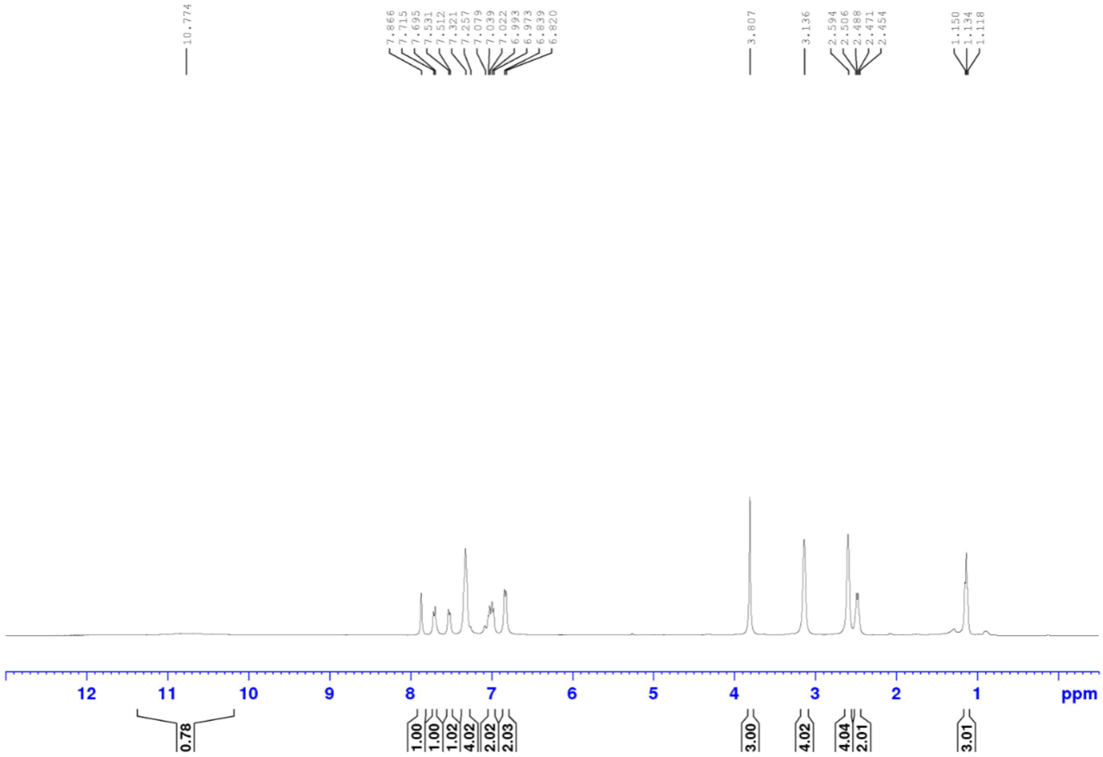

10.774
7.866
7.715
7.695
7.531
7.512
7.321
7.257
7.079
7.039
7.022
6.993
6.973
6.839
6.820
3.807
3.136
2.594
2.506
2.488
2.471
2.454
1.150
1.134
1.118
12 11 10 9 8 7 6 5 4 3 2 1 ppm
0.78
1.00 1.00 1.02 4.02 2.02 2.03
3.00
4.02
4.04 2.01
3.01


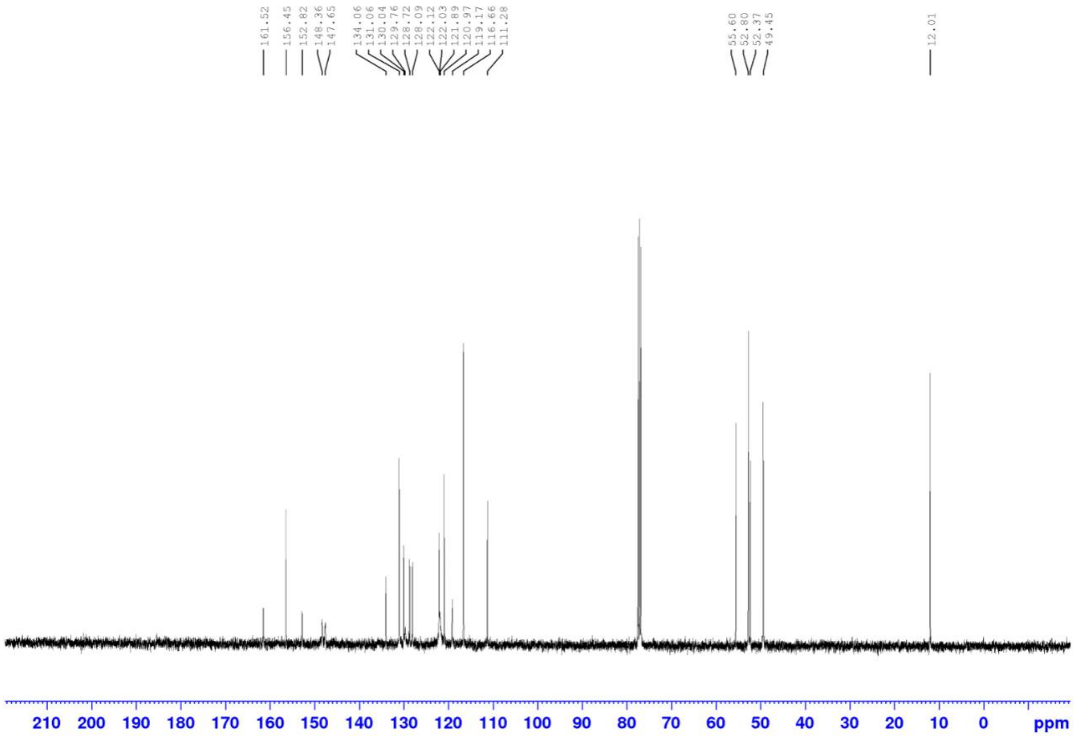

161.52
156.45
152.82
148.36
147.65
134.06
131.06
130.04
129.76
128.72
128.09
122.12
122.03
121.89
120.97
119.17
116.66
111.28
55.60
52.80
52.37
49.45
12.01
210 200 190 180 170 160 150 140 130 120 110 100 90 80 70 60 50 40 30 20 10 0 ppm

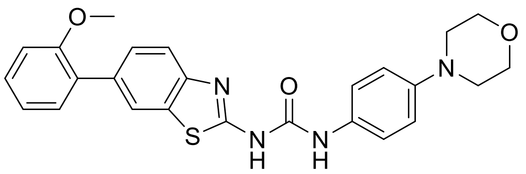
O
N
S
N
H
O
N
H
N
O

2c

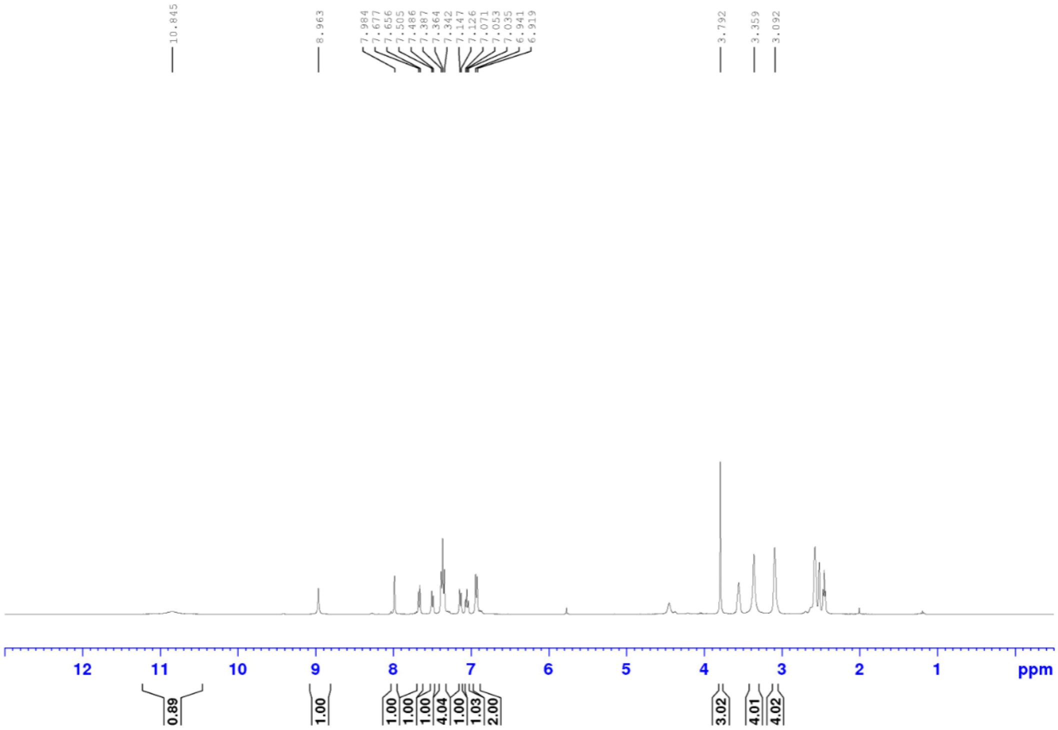
10.845
8.963
7.984
7.677
7.656
7.505
7.486
7.387
7.364
7.342
7.147
7.126
7.071
7.053
7.035
6.941
6.919
3.792
3.359
3.092
12 11 10 9 8 7 6 5 4 3 2 1 ppm
0.89
1.00
1.00
1.00
1.00
4.04
1.00
1.03
2.00
3.02
4.01
4.02

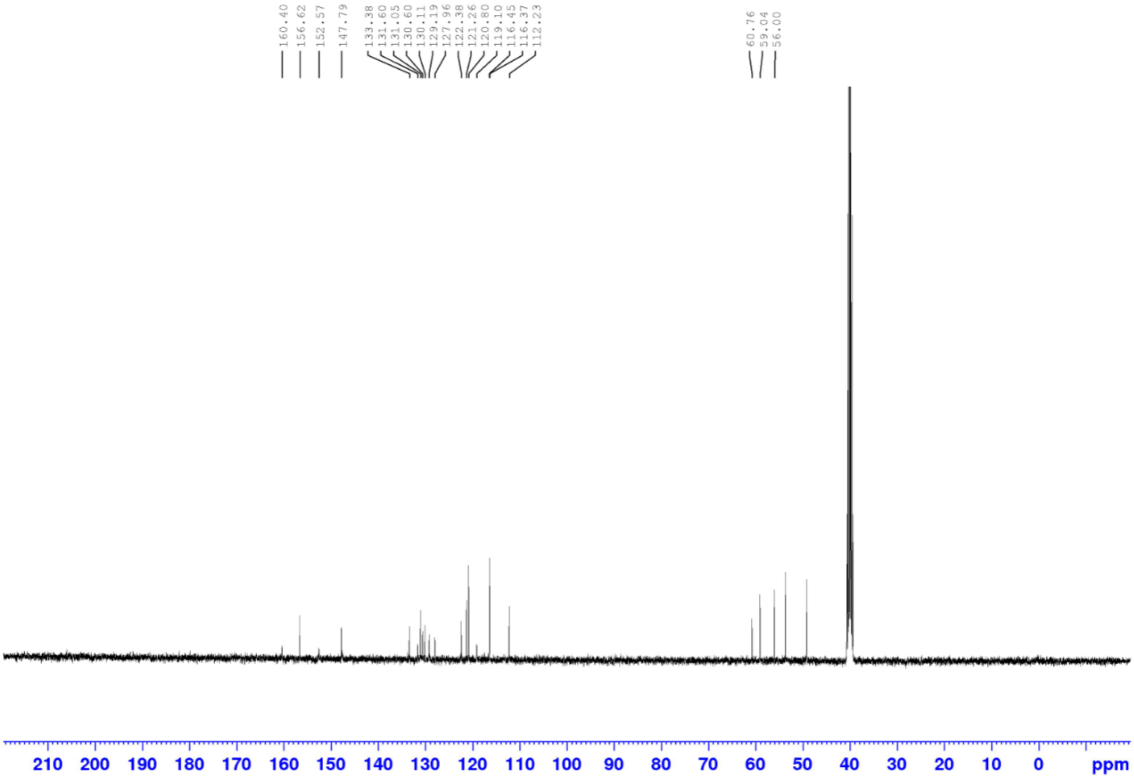
160.40
156.62
152.57
147.79
133.38
131.60
131.05
130.60
130.11
129.19
127.96
122.38
121.26
120.80
119.10
116.45
116.37
112.23
60.76
59.04
56.00
210 200 190 180 170 160 150 140 130 120 110 100 90 80 70 60 50 40 30 20 10 0 ppm

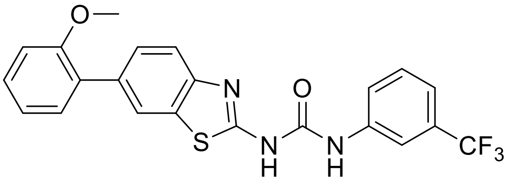

2d

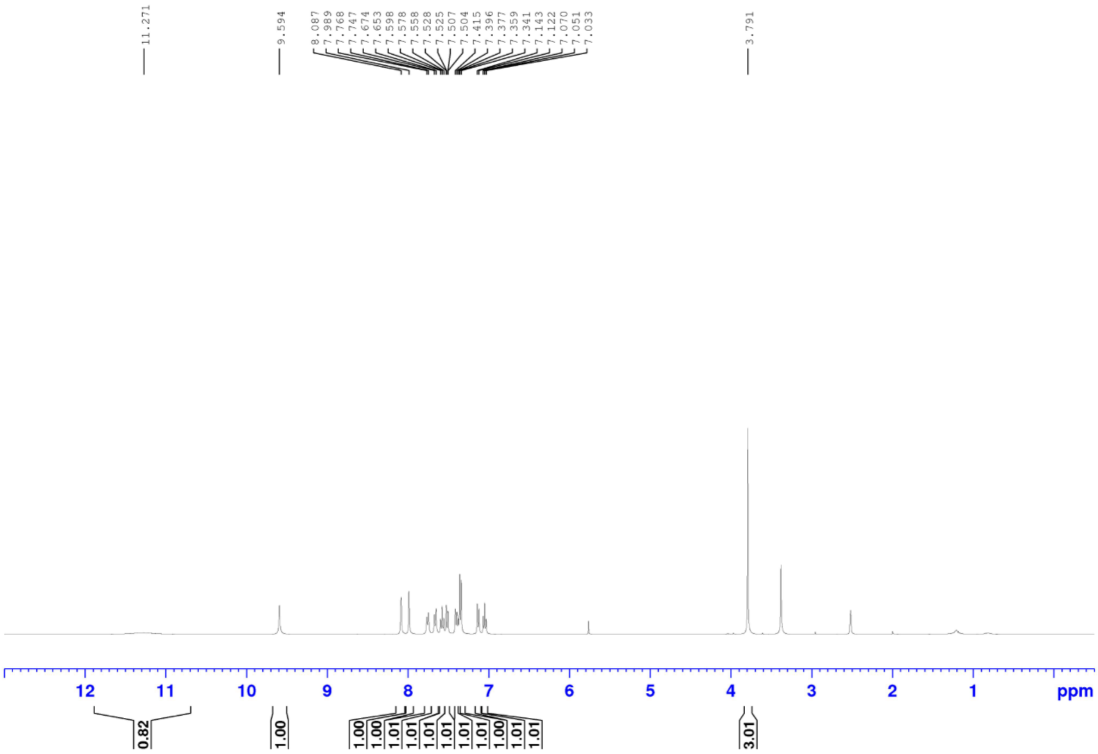
11.271
9.594
8.087 7.989 7.768 7.747 7.674 7.653 7.598 7.578 7.558 7.528 7.525 7.507 7.504 7.415 7.396 7.377 7.359 7.341 7.143 7.122 7.070 7.051 7.033
3.791
12 11 10 9 8 7 6 5 4 3 2 1 ppm
0.82
1.00
1.00 1.00 1.01 1.01 1.01 1.01 1.01 1.01 1.00 1.01 1.01
3.01

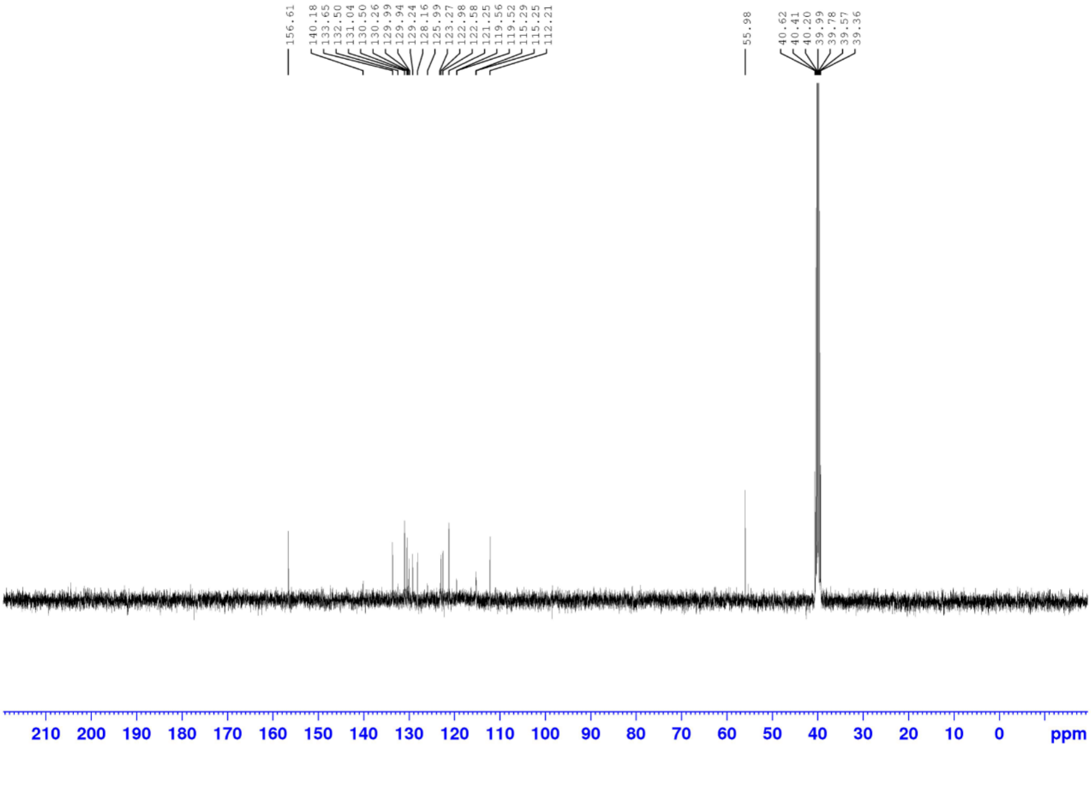
156.61
140.18 133.65 132.50 131.04 130.50 130.26 129.99 129.94 129.24 128.16 125.99 123.27 122.98 122.58 121.25 119.56 119.52 115.29 115.25 112.21
55.98
40.62 40.41 40.20 39.99 39.78 39.57 39.36
210 200 190 180 170 160 150 140 130 120 110 100 90 80 70 60 50 40 30 20 10 0 ppm

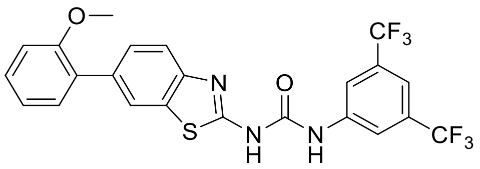
O
CF3
N
O
S
N
H
N
H
CF3

2e

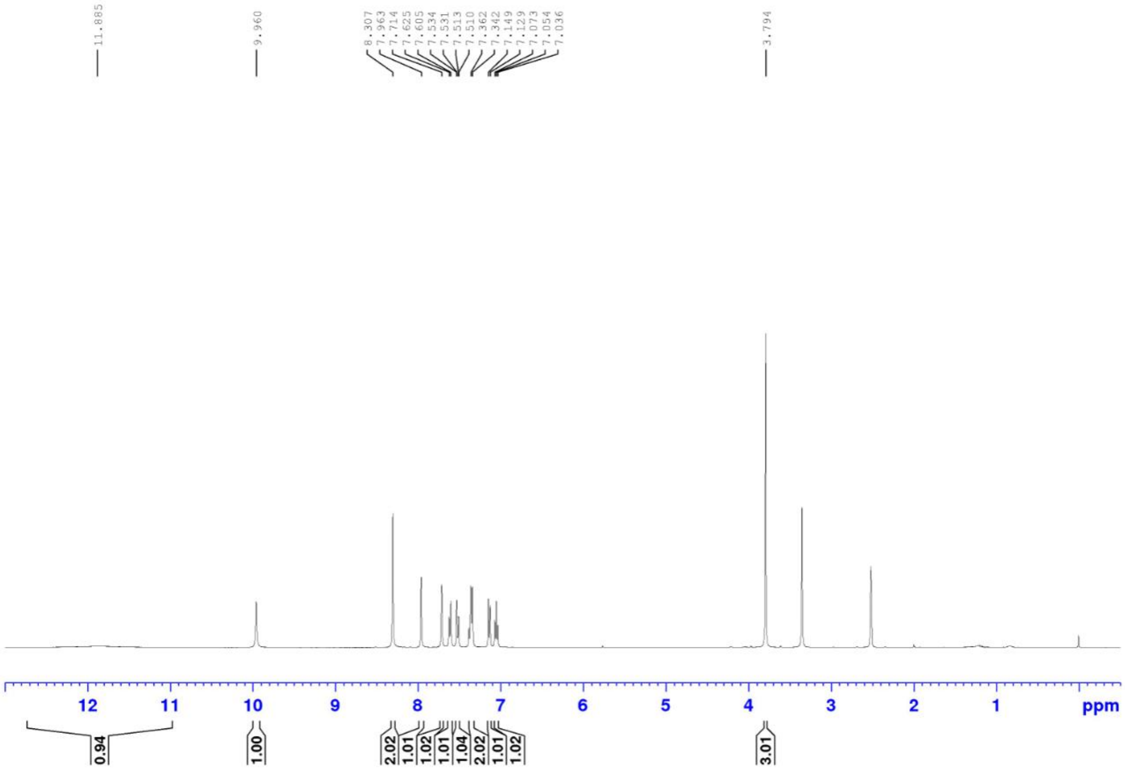
11.885
9.960
8.307
7.963
7.714
7.625
7.605
7.534
7.531
7.513
7.510
7.362
7.342
7.149
7.129
7.073
7.054
7.036
3.794
12 11 10 9 8 7 6 5 4 3 2 1 ppm
0.94
1.00
2.02
1.01
1.02
1.01
1.04
2.02
1.01
1.02
3.01

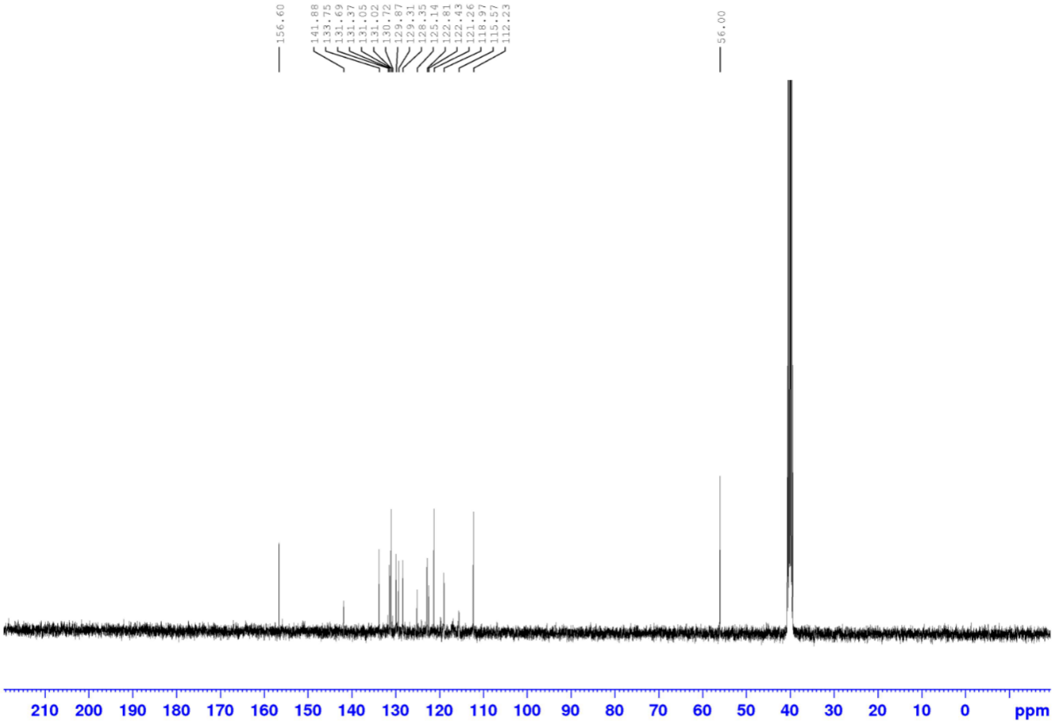
156.60
141.88
133.75
131.69
131.37
131.05
131.02
130.72
129.87
129.31
128.35
125.14
122.81
122.43
121.26
118.97
115.57
112.23
56.00
210 200 190 180 170 160 150 140 130 120 110 100 90 80 70 60 50 40 30 20 10 0 ppm

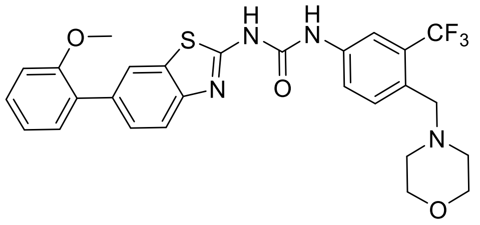

2f

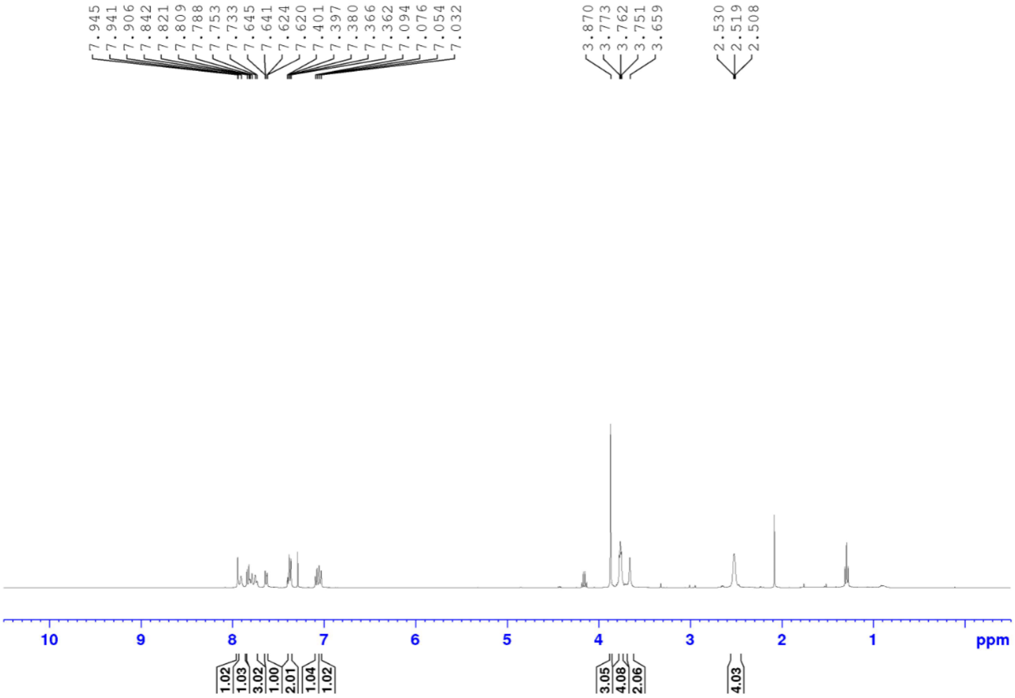

7.945
7.941
7.906
7.842
7.821
7.809
7.788
7.753
7.733
7.645
7.641
7.624
7.620
7.401
7.397
7.380
7.366
7.362
7.094
7.076
7.054
7.032
3.870
3.773
3.762
3.751
3.659
2.530
2.519
2.508
10
9
8
7
6
5
4
3
2
1
ppm
1.02
1.03
3.02
1.00
2.01
1.04
1.02
3.05
4.08
2.06
4.03


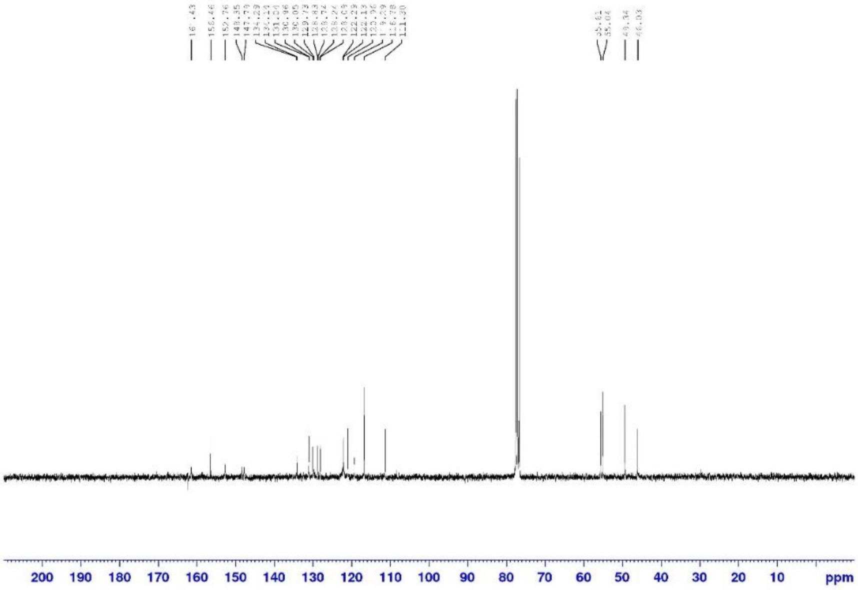

156.46
152.76
148.35
147.79
134.29
130.05
129.73
128.83
122.29
111.30
55.04
49.34
46.03
200
190
180
170
160
150
140
130
120
110
100
90
80
70
60
50
40
30
20
10
ppm

### 3) HRMS charts

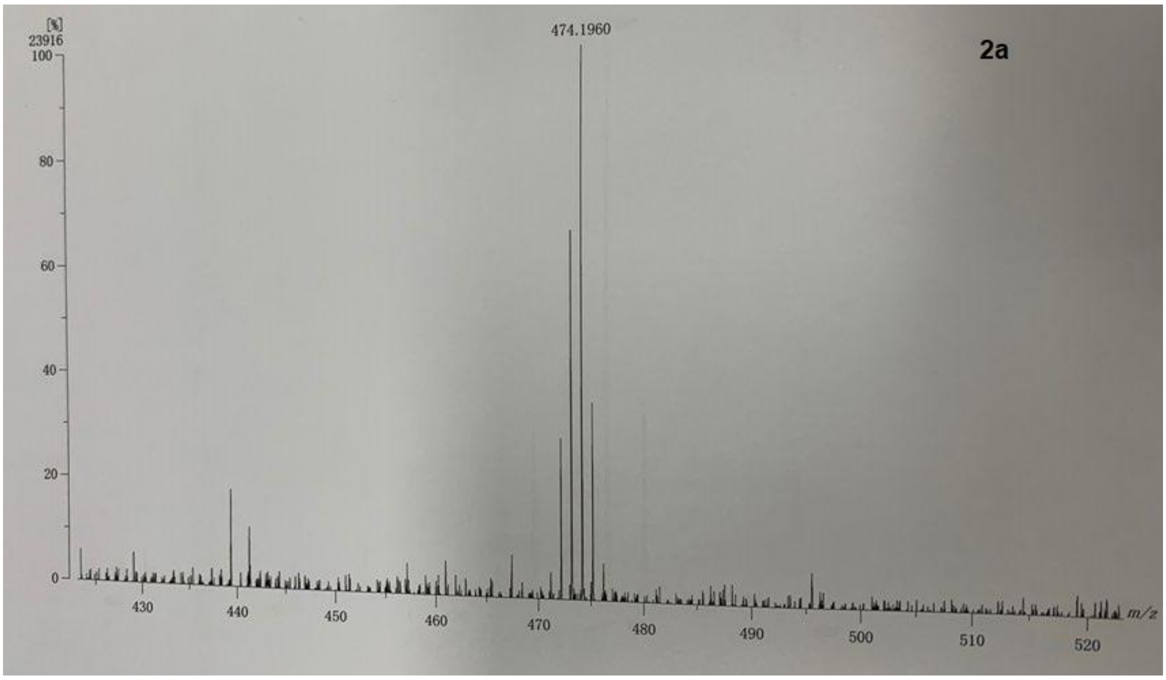


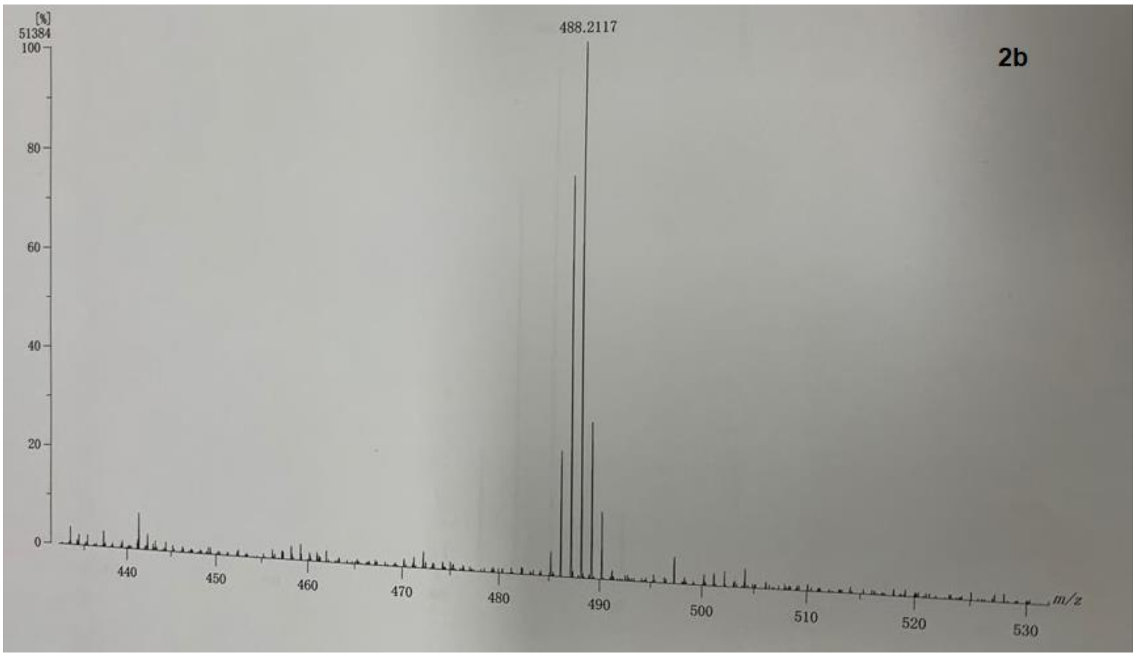

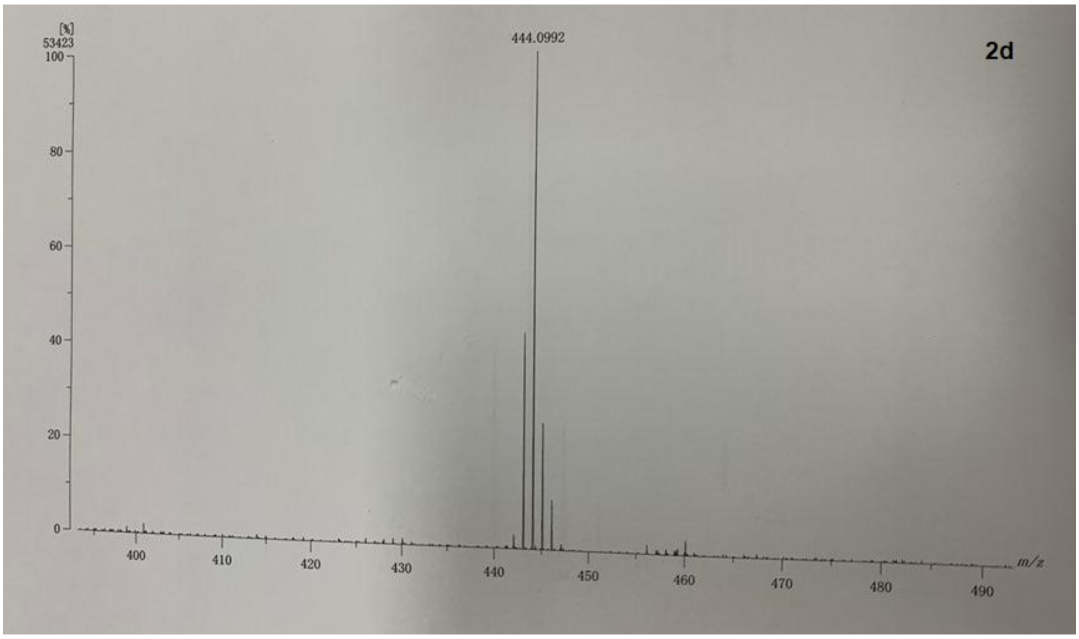
2d
[%]
53423
444.0992
m/z

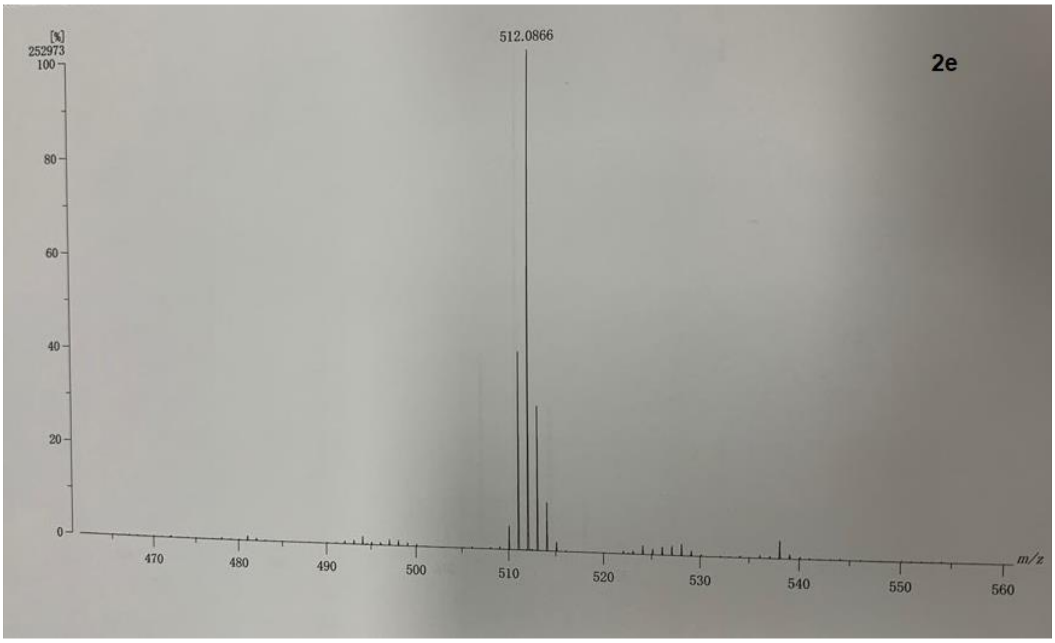
2e
[%]
252973
512.0866
m/z

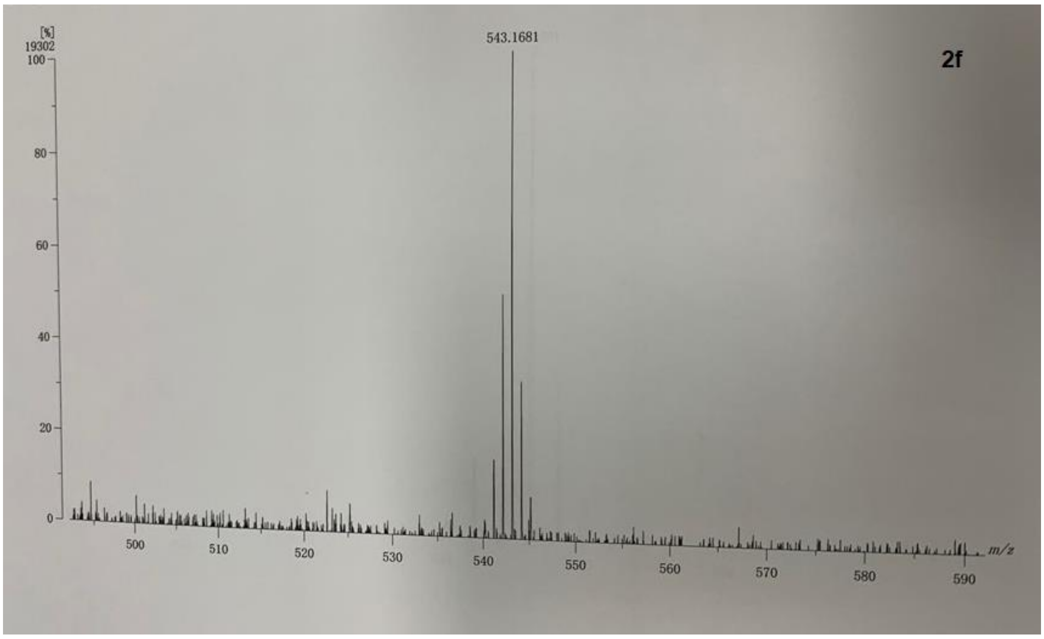
[%]
19302
100
80
60
40
20
0
543.1681
2f
500
510
520
530
540
550
560
570
580
590
m/z

### 4) 2D binding mode & bioavailability radar of 2b

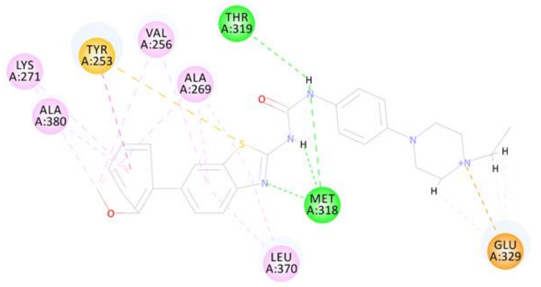


**2b/** BCR-ABL$^{WT}$

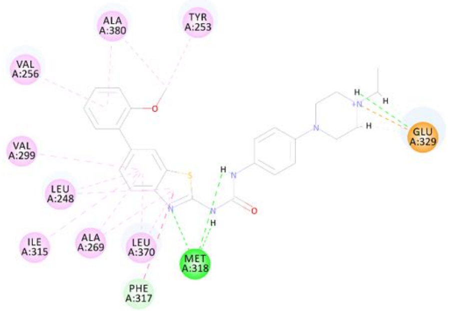


**2b/** BCR-ABL$^{T315I}$

**Figure S1**. 2D binding mode of compound **2b** with BCR-ABL$^{WT}$ and BCR-ABL$^{T315I}$. Various interactions are depicted by different color legends. The inhibitor is shown by line, interacting residues by colored sphere, and interactions by dash lines.

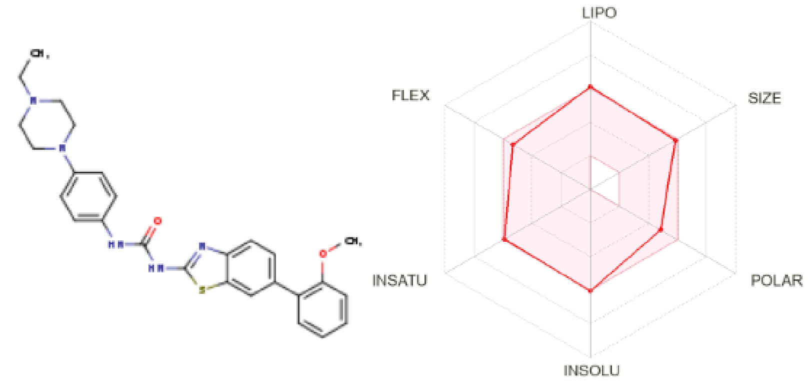


**Figure S2**. The bioavailability radar of **2b**.

## 5) NCI data

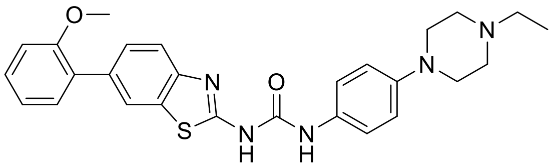

2b

| Panel/Cell Line | Growth Percent |
|---|---|
| **Leukemia** | |
| CCRF-CEM | -17.32 |
| HL-60(TB) | -74.14 |
| K-562 | -67.39 |
| MOLT-4 | -51.72 |
| RPMI-8226 | -32.99 |
| SR | -37.99 |
| **Non-Small Cell Lung Cancer** | |
| A549/ATCC | -77.13 |
| EKVX | -68.43 |
| HOP-62 | -66.15 |
| HOP-92 | 2.16 |
| NCI-H226 | 56.60 |
| NCI-H23 | -16.82 |
| NCI-H322M | -85.96 |
| NCI-H460 | -64.36 |
| NCI-H522 | -78.09 |
| **Colon Cancer** | |
| COLO 205 | -73.18 |
| HCC-2998 | -88.13 |
| HCT-116 | -82.33 |
| HCT-15 | -83.17 |
| HT29 | -92.03 |
| KM12 | -83.47 |
| SW-620 | -69.98 |
| **CNS Cancer** | |
| SF-268 | -59.43 |
| SF-295 | -79.95 |
| SF-539 | -82.57 |
| SNB-19 | -82.82 |
| SNB-75 | -19.81 |
| U251 | -86.98 |
| **Melanoma** | |
| LOX IMVI | -86.40 |
| MALME-3M | -80.30 |
| M14 | -70.13 |
| MDA-MB-435 | -85.49 |
| SK-MEL-2 | -65.48 |
| SK-MEL-28 | -79.30 |
| SK-MEL-5 | -98.79 |
| UACC-257 | -71.94 |
| UACC-62 | -85.26 |
| **Ovarian Cancer** | |
| IGROV1 | -71.35 |
| OVCAR-3 | -90.46 |
| OVCAR-4 | -76.12 |
| OVCAR-5 | -11.52 |
| OVCAR-8 | -52.55 |
| NCI/ADR-RES | -67.32 |
| SK-OV-3 | -12.52 |
| **Renal Cancer** | |
| 786-0 | -80.66 |
| A498 | 70.17 |
| ACHN | -95.48 |
| RXF 393 | -74.15 |
| SN12C | -82.09 |
| TK-10 | -48.01 |
| UO-31 | -91.59 |
| **Prostate Cancer** | |
| PC-3 | -83.78 |
| DU-145 | -84.44 |
| **Breast Cancer** | |
| MCF7 | -70.76 |
| MDA-MB-231/ATCC | -73.96 |
| HS 578T | -23.18 |
| BT-549 | 31.65 |
| T-47D | -45.42 |
| MDA-MB-468 | -76.67 |
| Mean | -60.96 |
| Delta | 37.83 |
| Range | 168.96 |

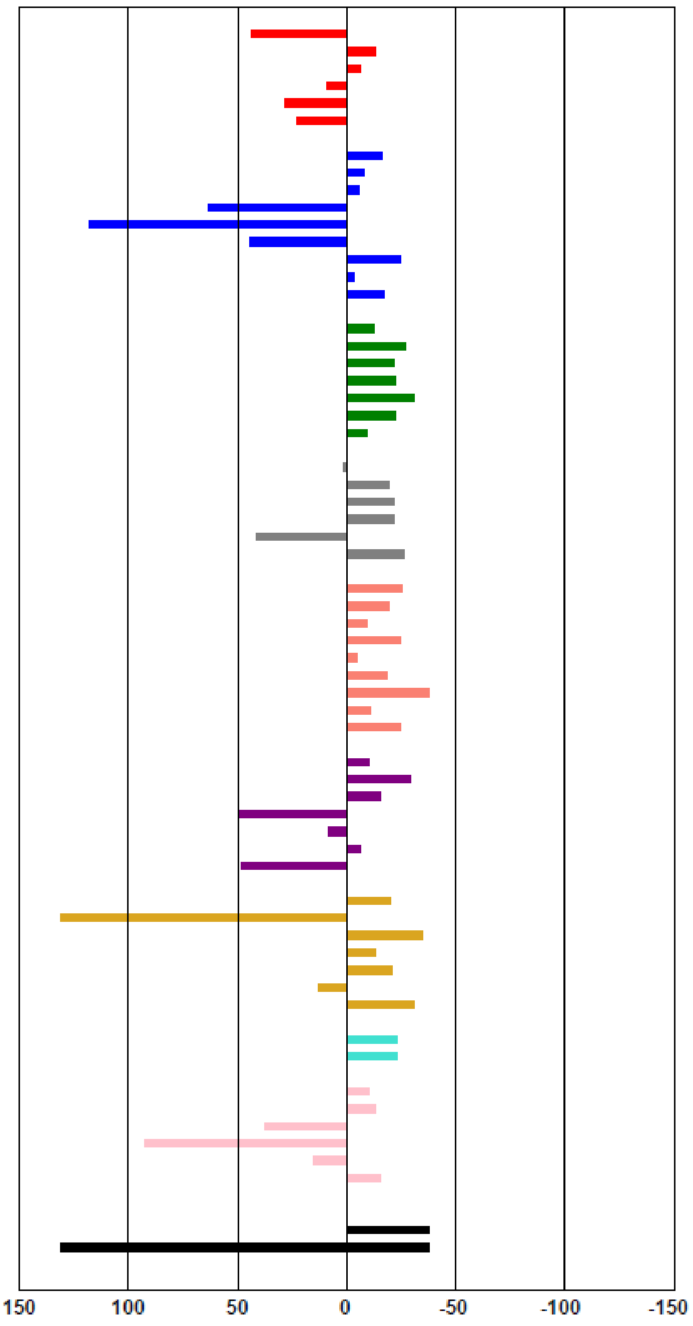

| Panel/Cell Line | Time Zero | Ctrl | Mean Optical Densities -8.0 | -7.0 | -6.0 | -5.0 | -4.0 | Percent Growth -8.0 | -7.0 | -6.0 | -5.0 | -4.0 | GI50 | TGI | LC50 |
|---|---|---|---|---|---|---|---|---|---|---|---|---|---|---|---|
| | | | Log10 Concentration | | | | | | | | | | | | |
| **Leukemia** | | | | | | | | | | | | | | | |
| CCRF-CEM | 0.623 | 2.866 | 2.809 | 2.829 | 2.564 | 0.282 | 0.220 | 97 | 98 | 87 | -55 | -65 | 1.81E-6 | 4.09E-6 | 9.25E-6 |
| HL-60(TB) | 0.842 | 2.792 | 2.774 | 2.804 | 2.553 | 0.246 | 0.269 | 99 | 101 | 88 | -71 | -68 | 1.73E-6 | 3.58E-6 | 7.39E-6 |
| K-562 | 0.226 | 2.152 | 2.181 | 1.885 | 0.397 | 0.088 | 0.137 | 101 | 86 | 9 | -61 | -40 | 2.93E-7 | 1.34E-6 | . |
| MOLT-4 | 0.803 | 2.963 | 2.942 | 3.017 | 2.783 | 0.235 | 0.268 | 99 | 103 | 92 | -71 | -67 | 1.80E-6 | 3.67E-6 | 7.45E-6 |
| RPMI-8226 | 0.767 | 2.614 | 2.709 | 2.712 | 2.517 | 0.397 | 0.318 | 105 | 105 | 95 | -48 | -59 | 2.06E-6 | 4.60E-6 | 1.48E-5 |
| SR | 0.367 | 1.200 | 1.123 | 1.150 | 1.001 | 0.206 | 0.227 | 91 | 94 | 76 | -44 | -38 | 1.65E-6 | 4.31E-6 | > 1.00E-4 |
| **Non-Small Cell Lung Cancer** | | | | | | | | | | | | | | | |
| A549/ATCC | 0.524 | 2.482 | 2.454 | 2.560 | 2.628 | 0.073 | 0.036 | 99 | 104 | 107 | -86 | -93 | 1.98E-6 | 3.59E-6 | 6.51E-6 |
| EKVX | 0.747 | 2.375 | 2.146 | 2.157 | 2.028 | 0.147 | 0.139 | 86 | 87 | 79 | -80 | -81 | 1.51E-6 | 3.12E-6 | 6.44E-6 |
| HOP-92 | 1.233 | 1.968 | 1.893 | 1.873 | 1.752 | 0.586 | 0.406 | 90 | 87 | 71 | -52 | -67 | 1.47E-6 | 3.75E-6 | 9.55E-6 |
| NCI-H226 | 1.055 | 2.311 | 2.314 | 2.328 | 2.283 | 0.512 | 0.415 | 100 | 101 | 98 | -52 | -61 | 2.09E-6 | 4.52E-6 | 9.77E-6 |
| NCI-H23 | 0.661 | 2.162 | 2.091 | 2.004 | 1.909 | 0.233 | 0.076 | 95 | 90 | 83 | -65 | -89 | 1.67E-6 | 3.65E-6 | 7.94E-6 |
| NCI-H322M | 1.063 | 2.402 | 2.367 | 2.234 | 2.149 | 0.133 | -0.020 | 97 | 87 | 81 | -87 | -100 | 1.53E-6 | 3.03E-6 | 5.99E-6 |
| NCI-H460 | 0.337 | 3.047 | 3.123 | 3.194 | 2.965 | 0.028 | -0.006 | 103 | 105 | 97 | -92 | -100 | 1.77E-6 | 3.27E-6 | 6.01E-6 |
| NCI-H522 | 1.256 | 2.983 | 2.765 | 2.786 | 2.812 | 0.308 | 0.196 | 87 | 89 | 90 | -76 | -84 | 1.75E-6 | 3.50E-6 | 7.01E-6 |
| **Colon Cancer** | | | | | | | | | | | | | | | |
| COLO 205 | 0.670 | 2.527 | 2.411 | 2.504 | 2.539 | 0.192 | 0.104 | 94 | 99 | 101 | -71 | -85 | 1.97E-6 | 3.85E-6 | 7.51E-6 |
| HCC-2998 | 0.631 | 2.454 | 2.350 | 2.366 | 2.430 | 0.080 | 0.036 | 94 | 95 | 99 | -87 | -94 | 1.83E-6 | 3.39E-6 | 6.30E-6 |
| HCT-116 | 0.243 | 2.129 | 2.110 | 2.193 | 1.862 | 0.016 | -0.022 | 99 | 103 | 86 | -94 | -100 | 1.58E-6 | 3.01E-6 | 5.71E-6 |
| HCT-15 | 0.225 | 1.765 | 1.603 | 1.628 | 1.195 | 0.006 | -0.043 | 89 | 91 | 63 | -98 | -100 | 1.20E-6 | 2.47E-6 | 5.06E-6 |
| HT29 | 0.362 | 2.517 | 2.453 | 2.369 | 1.842 | 0.035 | 0.002 | 97 | 93 | 69 | -90 | -100 | 1.31E-6 | 2.70E-6 | 5.57E-6 |
| KM12 | 0.508 | 2.744 | 2.797 | 2.812 | 2.604 | 0.073 | 0.067 | 102 | 103 | 94 | -86 | -87 | 1.75E-6 | 3.33E-6 | 6.33E-6 |
| SW-620 | 0.331 | 2.097 | 2.089 | 2.223 | 2.189 | 0.046 | 0.008 | 100 | 107 | 105 | -86 | -98 | 1.94E-6 | 3.55E-6 | 6.48E-6 |
| **CNS Cancer** | | | | | | | | | | | | | | | |
| SF-268 | 0.709 | 2.297 | 2.189 | 2.198 | 2.256 | 0.166 | 0.156 | 93 | 94 | 97 | -77 | -78 | 1.87E-6 | 3.63E-6 | 7.03E-6 |
| SF-295 | 0.631 | 2.340 | 2.133 | 2.247 | 2.207 | 0.027 | 0.020 | 88 | 95 | 92 | -96 | -97 | 1.68E-6 | 3.09E-6 | 5.71E-6 |
| SF-539 | 0.873 | 2.525 | 2.346 | 2.386 | 2.306 | 0.059 | 0.017 | 89 | 92 | 87 | -93 | -98 | 1.60E-6 | 3.03E-6 | 5.75E-6 |
| SNB-19 | 0.758 | 2.517 | 2.535 | 2.624 | 2.691 | 0.111 | 0.022 | 101 | 106 | 110 | -85 | -97 | 2.03E-6 | 3.65E-6 | 6.59E-6 |
| SNB-75 | 0.792 | 1.768 | 1.604 | 1.640 | 1.582 | 0.027 | 0.009 | 83 | 87 | 81 | -97 | -99 | 1.49E-6 | 2.86E-6 | 5.46E-6 |
| U251 | 0.534 | 2.305 | 2.311 | 2.372 | 2.061 | 0.013 | -0.030 | 100 | 104 | 86 | -98 | -100 | 1.57E-6 | 2.94E-6 | 5.51E-6 |
| **Melanoma** | | | | | | | | | | | | | | | |
| LOX IMVI | 0.364 | 2.746 | 2.579 | 2.588 | 2.568 | 0.029 | 0.012 | 93 | 93 | 93 | -92 | -97 | 1.70E-6 | 3.17E-6 | 5.92E-6 |
| MALME-3M | 0.701 | 1.183 | 1.146 | 1.136 | 1.136 | 0.128 | 0.050 | 92 | 90 | 90 | -82 | -93 | 1.71E-6 | 3.34E-6 | 6.54E-6 |
| M14 | 0.514 | 1.903 | 1.867 | 1.891 | 1.748 | 0.023 | -0.006 | 97 | 99 | 89 | -96 | -100 | 1.62E-6 | 3.03E-6 | 5.66E-6 |
| MDA-MB-435 | 0.454 | 2.584 | 2.464 | 2.491 | 2.470 | 0.018 | -0.018 | 94 | 96 | 95 | -96 | -100 | 1.71E-6 | 3.14E-6 | 5.74E-6 |
| SK-MEL-2 | 1.090 | 2.445 | 2.410 | 2.484 | 2.491 | 0.195 | 0.232 | 97 | 103 | 103 | -82 | -79 | 1.94E-6 | 3.61E-6 | 6.71E-6 |
| SK-MEL-28 | 0.836 | 2.202 | 2.090 | 2.153 | 2.141 | 0.077 | -0.001 | 92 | 96 | 96 | -91 | -100 | 1.76E-6 | 3.26E-6 | 6.04E-6 |
| SK-MEL-5 | 0.816 | 3.329 | 3.238 | 3.339 | 3.235 | 0.012 | -0.027 | 96 | 100 | 96 | -99 | -100 | 1.73E-6 | 3.12E-6 | 5.63E-6 |
| UACC-257 | 1.087 | 2.269 | 2.266 | 2.393 | 2.433 | 0.188 | 0.109 | 100 | 110 | 114 | -83 | -90 | 2.11E-6 | 3.79E-6 | 6.81E-6 |
| UACC-62 | 0.929 | 2.892 | 2.879 | 2.802 | 2.912 | 0.122 | 0.034 | 99 | 95 | 101 | -87 | -96 | 1.87E-6 | 3.45E-6 | 6.36E-6 |
| **Ovarian Cancer** | | | | | | | | | | | | | | | |
| IGROV1 | 0.481 | 1.995 | 2.007 | 2.074 | 1.953 | 0.110 | 0.043 | 101 | 105 | 97 | -77 | -91 | 1.87E-6 | 3.61E-6 | 6.99E-6 |
| OVCAR-3 | 0.459 | 1.788 | 1.837 | 1.854 | 1.637 | 0.040 | 0.033 | 104 | 105 | 89 | -91 | -93 | 1.64E-6 | 3.11E-6 | 5.90E-6 |
| OVCAR-4 | 0.710 | 1.555 | 1.481 | 1.516 | 1.455 | 0.133 | 0.044 | 91 | 95 | 88 | -81 | -94 | 1.68E-6 | 3.31E-6 | 6.53E-6 |
| OVCAR-5 | 0.789 | 1.857 | 1.664 | 1.793 | 1.820 | 0.108 | 0.017 | 82 | 94 | 97 | -86 | -98 | 1.80E-6 | 3.37E-6 | 6.33E-6 |
| OVCAR-8 | 0.593 | 2.439 | 2.483 | 2.635 | 2.558 | 0.105 | 0.061 | 102 | 111 | 106 | -82 | -90 | 1.99E-6 | 3.66E-6 | 6.74E-6 |
| NCI/ADR-RES | 0.529 | 2.011 | 1.985 | 1.933 | 1.867 | 0.098 | 0.058 | 98 | 95 | 90 | -82 | -89 | 1.72E-6 | 3.35E-6 | 6.55E-6 |
| SK-OV-3 | 1.180 | 2.536 | 2.431 | 2.492 | 2.433 | 0.290 | -0.007 | 92 | 97 | 92 | -75 | -100 | 1.79E-6 | 3.55E-6 | 7.05E-6 |
| **Renal Cancer** | | | | | | | | | | | | | | | |
| 786-0 | 0.745 | 2.694 | 2.581 | 2.728 | 2.681 | 0.050 | 0.042 | 94 | 102 | 99 | -93 | -94 | 1.80E-6 | 3.28E-6 | 5.96E-6 |
| A498 | 1.461 | 2.128 | 2.090 | 2.098 | 2.074 | 0.397 | 0.153 | 94 | 96 | 92 | -73 | -90 | 1.80E-6 | 3.61E-6 | 7.27E-6 |
| ACHN | 0.385 | 1.586 | 1.563 | 1.642 | 1.554 | -0.008 | -0.031 | 98 | 105 | 97 | -100 | -100 | 1.74E-6 | 3.11E-6 | 5.58E-6 |
| RXF 393 | 0.888 | 1.518 | 1.544 | 1.561 | 1.456 | 0.145 | 0.049 | 104 | 107 | 90 | -84 | -94 | 1.70E-6 | 3.30E-6 | 6.40E-6 |
| SN12C | 0.554 | 2.316 | 2.273 | 2.336 | 2.221 | 0.084 | 0.051 | 98 | 101 | 95 | -85 | -91 | 1.77E-6 | 3.37E-6 | 6.40E-6 |
| TK-10 | 0.939 | 1.899 | 1.825 | 1.856 | 2.103 | 0.032 | 0.010 | 92 | 96 | 121 | -97 | -99 | 2.12E-6 | 3.60E-6 | 6.11E-6 |
| UO-31 | 0.610 | 1.813 | 1.653 | 1.625 | 1.593 | 0.038 | 0.020 | 87 | 84 | 82 | -94 | -97 | 1.52E-6 | 2.92E-6 | 5.63E-6 |
| **Prostate Cancer** | | | | | | | | | | | | | | | |
| PC-3 | 0.671 | 2.722 | 2.638 | 2.637 | 2.598 | 0.136 | 0.111 | 96 | 96 | 94 | -80 | -84 | 1.79E-6 | 3.47E-6 | 6.74E-6 |
| DU-145 | 0.393 | 1.779 | 1.811 | 1.812 | 1.705 | 0.040 | -0.001 | 102 | 102 | 95 | -90 | -100 | 1.74E-6 | 3.26E-6 | 6.08E-6 |
| **Breast Cancer** | | | | | | | | | | | | | | | |
| MCF7 | 0.440 | 2.461 | 2.172 | 2.210 | 1.794 | 0.088 | 0.063 | 86 | 88 | 67 | -80 | -86 | 1.31E-6 | 2.86E-6 | 6.25E-6 |
| MDA-MB-231/ATCC | 0.663 | 2.145 | 2.152 | 2.137 | 2.021 | 0.094 | 0.096 | 100 | 99 | 92 | -86 | -86 | 1.72E-6 | 3.28E-6 | 6.28E-6 |
| HS 578T | 1.019 | 1.956 | 2.017 | 1.977 | 1.868 | 0.519 | 0.420 | 107 | 102 | 91 | -49 | -59 | 1.95E-6 | 4.45E-6 | 1.25E-5 |
| BT-549 | 1.227 | 2.206 | 2.022 | 2.186 | 2.171 | 0.343 | 0.196 | 81 | 98 | 96 | -72 | -84 | 1.88E-6 | 3.73E-6 | 7.39E-6 |
| T-47D | 0.976 | 2.089 | 1.949 | 2.018 | 1.842 | 0.373 | 0.304 | 87 | 94 | 78 | -62 | -69 | 1.58E-6 | 3.61E-6 | 8.23E-6 |
| MDA-MB-468 | 0.982 | 1.980 | 1.904 | 2.043 | 2.101 | 0.266 | 0.174 | 92 | 106 | 112 | -73 | -82 | 2.17E-6 | 4.03E-6 | 7.51E-6 |

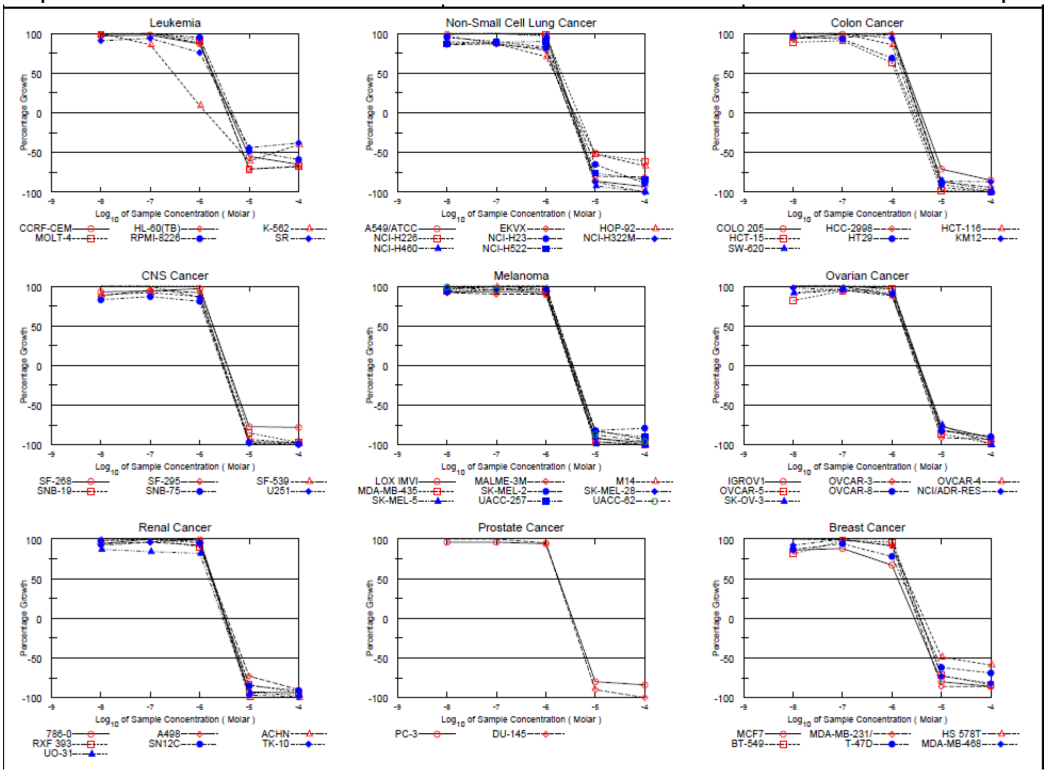

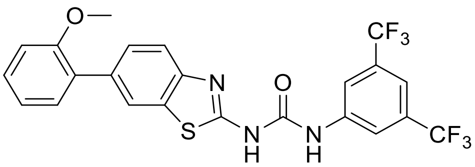

2e

| Panel/Cell Line | Growth Percent |
|---|---|
| Leukemia | |
| CCRF-CEM | 0.89 |
| HL-60(TB) | -34.47 |
| K-562 | 5.97 |
| MOLT-4 | -15.65 |
| RPMI-8226 | -15.91 |
| SR | -23.77 |
| Non-Small Cell Lung Cancer | |
| A549/ATCC | 12.73 |
| EKVX | 10.67 |
| HOP-62 | -1.58 |
| HOP-92 | -8.46 |
| NCI-H226 | 16.93 |
| NCI-H23 | 6.38 |
| NCI-H322M | 15.32 |
| NCI-H460 | -4.90 |
| NCI-H522 | -2.89 |
| Colon Cancer | |
| COLO 205 | -84.84 |
| HCC-2998 | 11.83 |
| HCT-116 | 4.85 |
| HCT-15 | 9.74 |
| HT29 | 4.76 |
| KM12 | 3.63 |
| SW-620 | 11.88 |
| CNS Cancer | |
| SF-268 | 16.82 |
| SF-295 | -0.29 |
| SF-539 | 5.20 |
| SNB-19 | 25.06 |
| SNB-75 | 8.11 |
| U251 | 12.49 |
| Melanoma | |
| LOX IMVI | 5.02 |
| MALME-3M | -13.04 |
| M14 | 16.03 |
| MDA-MB-435 | 10.94 |
| SK-MEL-2 | -14.06 |
| SK-MEL-28 | 31.11 |
| SK-MEL-5 | -86.07 |
| UACC-257 | -5.57 |
| UACC-62 | 10.85 |
| Ovarian Cancer | |
| IGROV1 | 42.38 |
| OVCAR-3 | 8.00 |
| OVCAR-4 | 16.54 |
| OVCAR-5 | 20.50 |
| OVCAR-8 | 9.40 |
| NCI/ADR-RES | 5.44 |
| SK-OV-3 | -13.23 |
| Renal Cancer | |
| 786-0 | 8.85 |
| A498 | -26.71 |
| ACHN | 8.64 |
| RXF 393 | -6.71 |
| SN12C | 13.03 |
| TK-10 | 9.11 |
| UO-31 | 13.14 |
| Prostate Cancer | |
| PC-3 | 13.52 |
| DU-145 | 19.48 |
| Breast Cancer | |
| MCF7 | 4.10 |
| MDA-MB-231/ATCC | -64.24 |
| HS 578T | -0.70 |
| BT-549 | 13.18 |
| T-47D | -13.55 |
| MDA-MB-468 | -8.23 |
| Mean | .30 |
| Delta | 86.37 |
| Range | 128.45 |

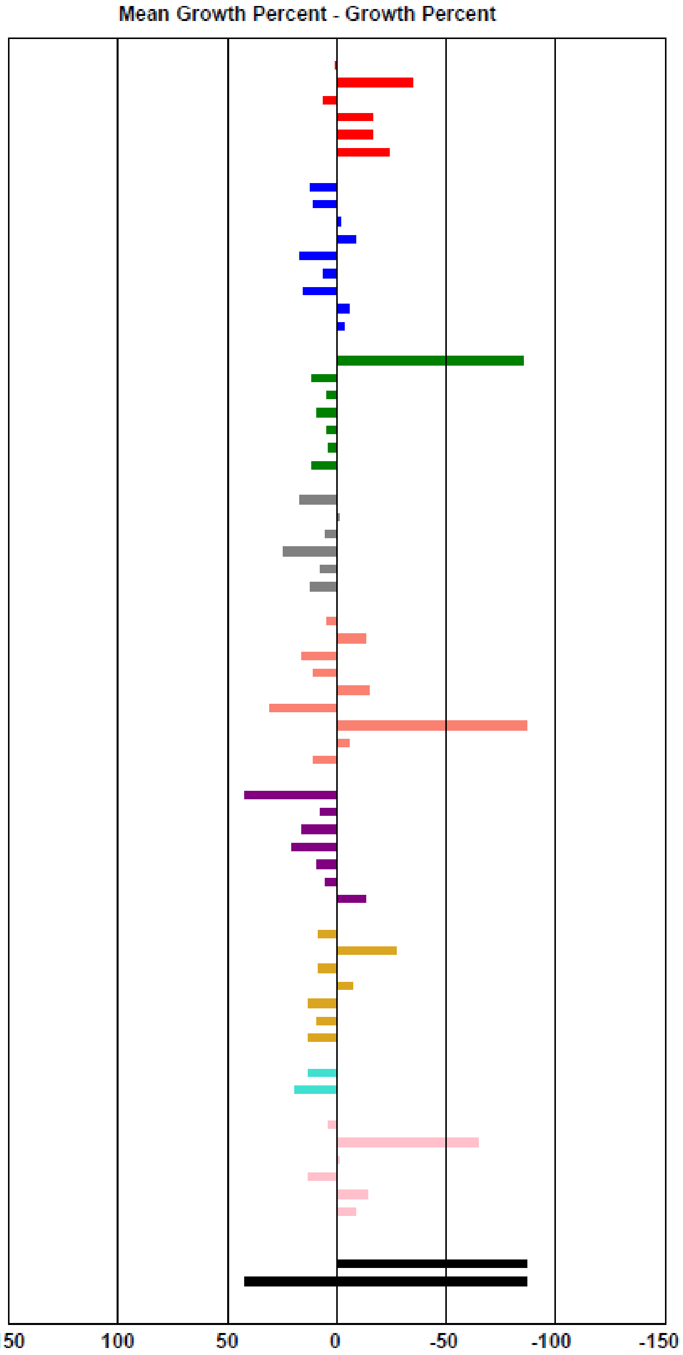

| | | | Log10 Concentration | | | | | | | | | | | | |
|---|---|---|---|---|---|---|---|---|---|---|---|---|---|---|---|
| | Time | | Mean Optical Densities | | | | | Percent Growth | | | | | | | |
| Panel/Cell Line | Zero | Ctrl | -8.0 | -7.0 | -6.0 | -5.0 | -4.0 | -8.0 | -7.0 | -6.0 | -5.0 | -4.0 | GI50 | TGI | LC50 |
| Leukemia | | | | | | | | | | | | | | | |
| CCRF-CEM | 0.434 | 1.384 | 1.500 | 1.548 | 1.154 | 0.518 | 0.255 | 112 | 117 | 76 | 9 | -41 | 2.43E-6 | 1.50E-5 | > 1.00E-4 |
| HL-60(TB) | 0.750 | 1.867 | 1.687 | 1.749 | 1.790 | 0.384 | 0.112 | 84 | 89 | 93 | -49 | -85 | 2.01E-6 | 4.53E-6 | 1.08E-5 |
| K-562 | 0.269 | 1.586 | 1.495 | 1.380 | 1.089 | 0.261 | 0.065 | 93 | 84 | 62 | -3 | -76 | 1.54E-6 | 8.95E-6 | 4.39E-5 |
| MOLT-4 | 0.735 | 2.182 | 1.850 | 1.837 | 1.678 | 0.657 | 0.250 | 77 | 76 | 65 | -11 | -66 | 1.59E-6 | 7.24E-6 | 5.13E-5 |
| RPMI-8226 | 0.587 | 2.118 | 2.189 | 2.289 | 1.647 | 0.459 | 0.220 | 105 | 111 | 69 | -22 | -63 | 1.63E-6 | 5.76E-6 | 4.93E-5 |
| SR | 0.441 | 1.333 | 1.266 | 1.158 | 1.118 | 0.311 | 0.071 | 92 | 80 | 76 | -29 | -84 | 1.76E-6 | 5.25E-6 | 2.38E-5 |
| Non-Small Cell Lung Cancer | | | | | | | | | | | | | | | |
| A549/ATCC | 0.361 | 1.574 | 1.583 | 1.554 | 1.506 | 0.437 | 0.070 | 101 | 98 | 94 | 6 | -81 | 3.19E-6 | 1.18E-5 | 4.43E-5 |
| HOP-62 | 0.745 | 2.019 | 1.897 | 1.924 | 1.931 | 0.445 | 0.020 | 90 | 93 | 93 | -40 | -97 | 2.10E-6 | 4.99E-6 | 1.48E-5 |
| HOP-92 | 1.114 | 1.611 | 1.506 | 1.512 | 1.396 | 0.750 | 0.123 | 79 | 80 | 57 | -33 | -89 | 1.19E-6 | 4.31E-6 | 2.03E-5 |
| NCI-H226 | 0.841 | 1.812 | 1.788 | 1.838 | 1.714 | 0.594 | 0.127 | 98 | 103 | 90 | -29 | -85 | 2.16E-6 | 5.67E-6 | 2.35E-5 |
| NCI-H23 | 0.511 | 1.811 | 1.795 | 1.743 | 1.524 | 0.441 | 0.013 | 99 | 95 | 78 | -14 | -98 | 2.02E-6 | 7.09E-6 | 2.71E-5 |
| NCI-H322M | 0.874 | 2.013 | 1.895 | 1.888 | 1.937 | 0.838 | 0.133 | 90 | 89 | 93 | -4 | -85 | 2.78E-6 | 9.06E-6 | 3.70E-5 |
| NCI-H460 | 0.221 | 2.147 | 2.227 | 2.333 | 1.971 | 0.167 | 0.016 | 104 | 110 | 91 | -25 | -93 | 2.26E-6 | 6.12E-6 | 2.35E-5 |
| NCI-H522 | 0.892 | 2.064 | 1.858 | 1.841 | 1.676 | 0.874 | 0.068 | 82 | 81 | 67 | -2 | -92 | 1.76E-6 | 9.35E-6 | 3.39E-5 |
| Colon Cancer | | | | | | | | | | | | | | | |
| COLO 205 | 0.508 | 1.825 | 1.786 | 1.825 | 1.551 | 0.232 | 0.034 | 97 | 100 | 79 | -54 | -93 | 1.65E-6 | 3.92E-6 | 9.28E-6 |
| HCC-2998 | 0.694 | 2.320 | 2.287 | 2.188 | 2.051 | 0.767 | 0.047 | 98 | 92 | 83 | 4 | -93 | 2.65E-6 | 1.11E-5 | 3.61E-5 |
| HCT-116 | 0.195 | 1.417 | 1.188 | 1.313 | 0.854 | 0.030 | 0.008 | 81 | 92 | 54 | -85 | -96 | 1.07E-6 | 2.45E-6 | 5.61E-6 |
| HCT-15 | 0.192 | 1.373 | 1.263 | 1.309 | 1.166 | 0.223 | 0.010 | 91 | 95 | 82 | 3 | -95 | 2.55E-6 | 1.06E-5 | 3.46E-5 |
| HT29 | 0.230 | 1.350 | 1.332 | 1.275 | 1.084 | 0.224 | 0.014 | 98 | 93 | 76 | -3 | -94 | 2.15E-6 | 9.21E-6 | 3.30E-5 |
| KM12 | 0.508 | 2.595 | 2.579 | 2.549 | 2.217 | 0.410 | 0.032 | 99 | 98 | 82 | -19 | -94 | 2.07E-6 | 6.45E-6 | 2.59E-5 |
| SW-620 | 0.265 | 1.762 | 1.779 | 1.817 | 1.657 | 0.327 | 0.053 | 101 | 104 | 93 | 4 | -80 | 3.04E-6 | 1.12E-5 | 4.40E-5 |
| CNS Cancer | | | | | | | | | | | | | | | |
| SF-268 | 0.537 | 1.918 | 1.909 | 1.854 | 1.704 | 0.660 | 0.040 | 99 | 95 | 85 | 9 | -93 | 2.86E-6 | 1.22E-5 | 3.81E-5 |
| SF-295 | 0.604 | 2.296 | 2.074 | 2.166 | 2.100 | 0.588 | 0.019 | 87 | 92 | 88 | -3 | -97 | 2.64E-6 | 9.33E-6 | 3.18E-5 |
| SF-539 | 1.012 | 2.615 | 2.324 | 2.446 | 2.357 | 0.714 | 0.010 | 82 | 89 | 84 | -29 | -99 | 1.99E-6 | 5.50E-6 | 1.97E-5 |
| SNB-19 | 0.445 | 1.798 | 1.795 | 1.818 | 1.470 | 0.570 | 0.013 | 100 | 101 | 76 | 9 | -97 | 2.44E-6 | 1.22E-5 | 3.61E-5 |
| SNB-75 | 0.886 | 1.716 | 1.563 | 1.647 | 1.499 | 0.938 | 0.182 | 82 | 92 | 74 | 6 | -79 | 2.25E-6 | 1.18E-5 | 4.53E-5 |
| U251 | 0.334 | 1.474 | 1.533 | 1.576 | 1.223 | 0.348 | 0.020 | 105 | 109 | 78 | 1 | -94 | 2.31E-6 | 1.03E-5 | 3.44E-5 |
| Melanoma | | | | | | | | | | | | | | | |
| LOX IMVI | 0.333 | 2.312 | 2.255 | 2.242 | 2.147 | 0.023 | 0.009 | 97 | 96 | 92 | -93 | -97 | 1.68E-6 | 3.13E-6 | 5.84E-6 |
| MALME-3M | 0.572 | 1.196 | 1.156 | 1.171 | 1.097 | 0.292 | 0.009 | 94 | 96 | 84 | -49 | -98 | 1.81E-6 | 4.29E-6 | 1.05E-5 |
| M14 | 0.420 | 1.512 | 1.338 | 1.408 | 1.289 | 0.097 | 0.006 | 84 | 90 | 80 | -77 | -99 | 1.54E-6 | 3.22E-6 | 6.73E-6 |
| MDA-MB-435 | 0.592 | 2.405 | 2.282 | 2.381 | 2.184 | 0.484 | 0.001 | 93 | 99 | 88 | -18 | -100 | 2.27E-6 | 6.73E-6 | 2.45E-5 |
| SK-MEL-2 | 1.031 | 2.081 | 2.030 | 2.023 | 1.917 | 0.792 | 0.024 | 95 | 95 | 84 | -23 | -98 | 2.09E-6 | 6.09E-6 | 2.29E-5 |
| SK-MEL-28 | 0.753 | 2.248 | 2.091 | 2.201 | 2.036 | 0.726 | 0.005 | 90 | 97 | 86 | -4 | -99 | 2.51E-6 | 9.10E-6 | 3.05E-5 |
| SK-MEL-5 | 0.632 | 2.939 | 2.848 | 2.917 | 2.601 | 0.033 | 0.009 | 96 | 99 | 85 | -95 | -99 | 1.57E-6 | 2.98E-6 | 5.64E-6 |
| UACC-257 | 1.062 | 1.992 | 1.973 | 2.015 | 1.901 | 0.844 | 0.034 | 98 | 102 | 90 | -21 | -97 | 2.31E-6 | 6.52E-6 | 2.43E-5 |
| UACC-62 | 0.631 | 2.445 | 2.333 | 2.455 | 2.091 | 0.149 | -0.005 | 94 | 101 | 80 | -76 | -100 | 1.56E-6 | 3.26E-6 | 6.78E-6 |
| Ovarian Cancer | | | | | | | | | | | | | | | |
| IGROV1 | 0.375 | 1.737 | 1.719 | 1.691 | 1.586 | 0.412 | 0.024 | 99 | 97 | 89 | 3 | -94 | 2.83E-6 | 1.07E-5 | 3.52E-5 |
| OVCAR-3 | 0.417 | 1.450 | 1.478 | 1.471 | 1.155 | 0.264 | 0.015 | 103 | 102 | 71 | -37 | -97 | 1.58E-6 | 4.58E-6 | 1.67E-5 |
| OVCAR-4 | 0.826 | 1.664 | 1.576 | 1.613 | 1.448 | 0.825 | 0.034 | 90 | 94 | 74 | 0 | -96 | 2.12E-6 | 9.96E-6 | 3.32E-5 |
| OVCAR-5 | 0.581 | 1.340 | 1.245 | 1.242 | 1.226 | 0.574 | 0.023 | 87 | 87 | 85 | -1 | -96 | 2.54E-6 | 9.66E-6 | 3.27E-5 |
| OVCAR-8 | 0.581 | 2.200 | 2.228 | 2.213 | 2.092 | 0.642 | 0.176 | 102 | 101 | 93 | 4 | -70 | 3.04E-6 | 1.12E-5 | 5.38E-5 |
| NCI/ADR-RES | 0.487 | 1.677 | 1.683 | 1.636 | 1.546 | 0.415 | 0.093 | 100 | 96 | 89 | -15 | -81 | 2.37E-6 | 7.19E-6 | 3.40E-5 |
| SK-OV-3 | 0.894 | 1.865 | 1.764 | 1.849 | 1.754 | 0.949 | 0.067 | 90 | 98 | 88 | 6 | -93 | 2.92E-6 | 1.14E-5 | 3.69E-5 |
| Renal Cancer | | | | | | | | | | | | | | | |
| 786-0 | 0.403 | 1.704 | 1.537 | 1.602 | 1.343 | 0.126 | 0.005 | 87 | 92 | 72 | -69 | -99 | 1.44E-6 | 3.25E-6 | 7.36E-6 |
| A498 | 1.422 | 2.138 | 2.082 | 2.081 | 1.987 | 0.645 | 0.014 | 92 | 92 | 79 | -55 | -99 | 1.64E-6 | 3.90E-6 | 9.23E-6 |
| ACHN | 0.379 | 1.616 | 1.521 | 1.612 | 1.364 | 0.341 | 0.006 | 92 | 100 | 80 | -10 | -99 | 2.14E-6 | 7.71E-6 | 2.82E-5 |
| CAKI-1 | 0.767 | 2.725 | 2.463 | 2.561 | 2.574 | 0.594 | 0.031 | 87 | 92 | 92 | -23 | -96 | 2.33E-6 | 6.35E-6 | 2.36E-5 |
| RXF 393 | 0.685 | 1.324 | 1.379 | 1.393 | 1.248 | 0.526 | 0.022 | 109 | 111 | 88 | -23 | -97 | 2.19E-6 | 6.18E-6 | 2.31E-5 |
| SN12C | 0.391 | 1.688 | 1.695 | 1.704 | 1.544 | 0.079 | 0.002 | 101 | 101 | 89 | -80 | -99 | 1.70E-6 | 3.36E-6 | 6.65E-6 |
| TK-10 | 0.865 | 1.691 | 1.602 | 1.595 | 1.762 | 0.962 | 0.117 | 89 | 88 | 109 | 12 | -87 | 4.03E-6 | 1.32E-5 | 4.25E-5 |
| UO-31 | 0.608 | 2.046 | 1.960 | 1.956 | 1.833 | 0.277 | 0.035 | 94 | 94 | 85 | -54 | -94 | 1.79E-6 | 4.07E-6 | 9.29E-6 |
| Prostate Cancer | | | | | | | | | | | | | | | |
| PC-3 | 0.440 | 1.367 | 1.323 | 1.361 | 1.023 | 0.388 | 0.023 | 95 | 99 | 63 | -12 | -95 | 1.49E-6 | 6.93E-6 | 2.88E-5 |
| DU-145 | 0.338 | 1.482 | 1.536 | 1.542 | 1.444 | 0.558 | 0.030 | 105 | 105 | 97 | 19 | -91 | 4.00E-6 | 1.49E-5 | 4.24E-5 |
| Breast Cancer | | | | | | | | | | | | | | | |
| MCF7 | 0.346 | 2.183 | 1.937 | 1.934 | 1.746 | 0.401 | 0.081 | 87 | 86 | 76 | 3 | -77 | 2.28E-6 | 1.09E-5 | 4.62E-5 |
| MDA-MB-231/ATCC | 0.547 | 1.431 | 1.428 | 1.460 | 1.367 | 0.246 | 0.019 | 100 | 103 | 93 | -55 | -97 | 1.95E-6 | 4.24E-6 | 9.23E-6 |
| HS 578T | 0.815 | 1.585 | 1.617 | 1.619 | 1.478 | 0.724 | 0.370 | 104 | 104 | 86 | -11 | -55 | 2.35E-6 | 7.67E-6 | 7.83E-5 |
| BT-549 | 1.139 | 1.947 | 1.789 | 1.840 | 1.777 | 0.463 | 0.007 | 80 | 87 | 79 | -59 | -99 | 1.62E-6 | 3.72E-6 | 8.55E-6 |
| T-47D | 0.703 | 1.578 | 1.451 | 1.490 | 1.361 | 0.701 | 0.396 | 85 | 90 | 75 | 0 | -44 | 2.16E-6 | 9.89E-6 | > 1.00E-4 |
| MDA-MB-468 | 0.640 | 1.326 | 1.337 | 1.353 | 1.095 | 0.487 | 0.095 | 102 | 104 | 66 | -24 | -85 | 1.52E-6 | 5.43E-6 | 2.67E-5 |

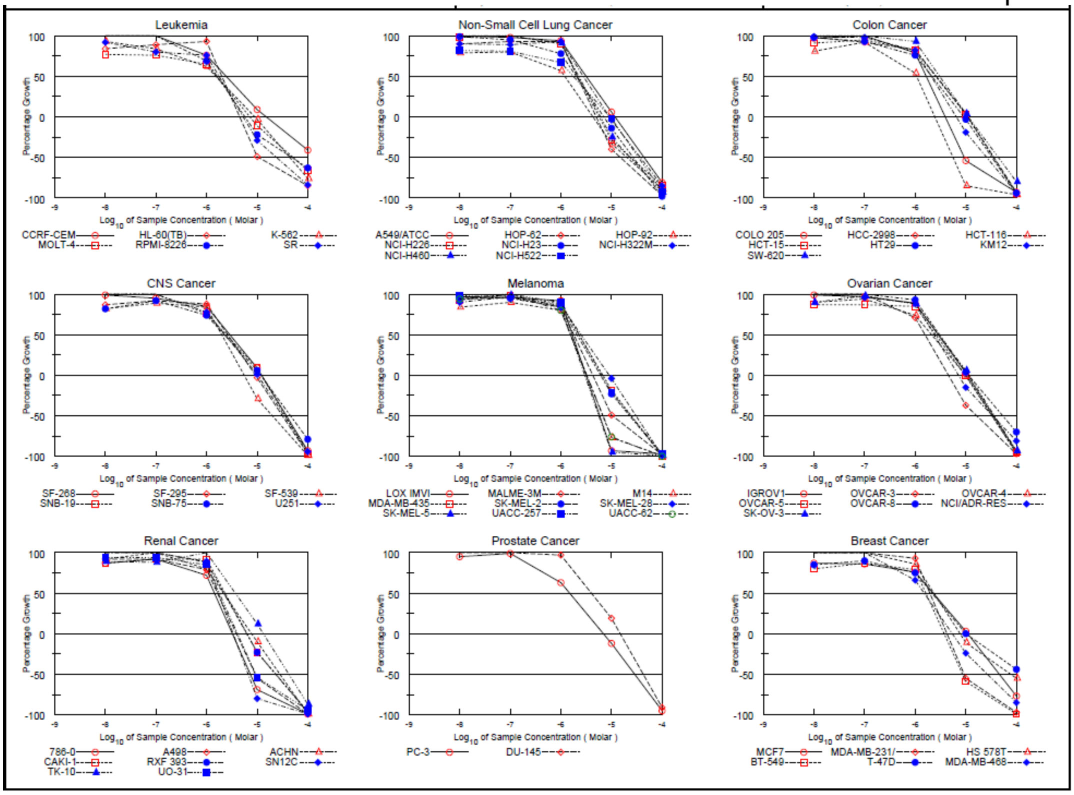

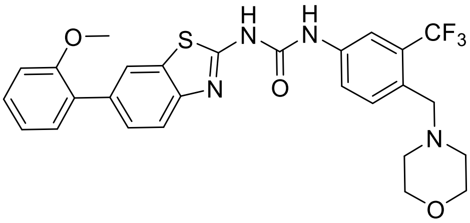

2f

| Panel/Cell Line | Growth Percent |
|---|---|
| Leukemia | |
| CCRF-CEM | 36.25 |
| HL-60(TB) | 34.35 |
| K-562 | 16.28 |
| MOLT-4 | 20.12 |
| RPMI-8226 | 21.02 |
| SR | 43.86 |
| Non-Small Cell Lung Cancer | |
| A549/ATCC | 56.73 |
| EKVX | 43.08 |
| HOP-62 | 85.54 |
| HOP-92 | 64.91 |
| NCI-H226 | 47.72 |
| NCI-H23 | 37.39 |
| NCI-H322M | 74.81 |
| NCI-H460 | 36.68 |
| NCI-H522 | 31.16 |
| Colon Cancer | |
| COLO 205 | 78.60 |
| HCC-2998 | 66.86 |
| HCT-116 | 32.14 |
| HCT-15 | 42.93 |
| HT29 | 48.73 |
| KM12 | 46.83 |
| SW-620 | 70.33 |
| CNS Cancer | |
| SF-268 | 71.62 |
| SF-295 | 49.07 |
| SF-539 | 70.30 |
| SNB-19 | 63.40 |
| SNB-75 | 62.53 |
| U251 | 48.57 |
| Melanoma | |
| LOX IMVI | 57.27 |
| MALME-3M | 80.24 |
| M14 | 58.96 |
| MDA-MB-435 | 73.41 |
| SK-MEL-2 | 71.59 |
| SK-MEL-28 | 79.06 |
| SK-MEL-5 | 54.29 |
| UACC-257 | 78.71 |
| UACC-62 | 58.65 |
| Ovarian Cancer | |
| IGROV1 | 71.54 |
| OVCAR-3 | 35.25 |
| OVCAR-4 | 46.05 |
| OVCAR-5 | 89.14 |
| OVCAR-8 | 49.98 |
| NCI/ADR-RES | 46.06 |
| SK-OV-3 | 94.11 |
| Renal Cancer | |
| 786-0 | 48.29 |
| A498 | 76.21 |
| ACHN | 52.13 |
| RXF 393 | 72.36 |
| SN12C | 61.42 |
| TK-10 | 60.13 |
| UO-31 | 56.97 |
| Prostate Cancer | |
| PC-3 | 34.90 |
| DU-145 | 56.86 |
| Breast Cancer | |
| MCF7 | 25.52 |
| MDA-MB-231/ATCC | 79.35 |
| HS 578T | 70.89 |
| BT-549 | 100.35 |
| T-47D | 32.01 |
| MDA-MB-468 | 39.93 |
| Mean | 56.16 |
| Delta | 39.88 |
| Range | 84.07 |

Mean Growth Percent - Growth Percent

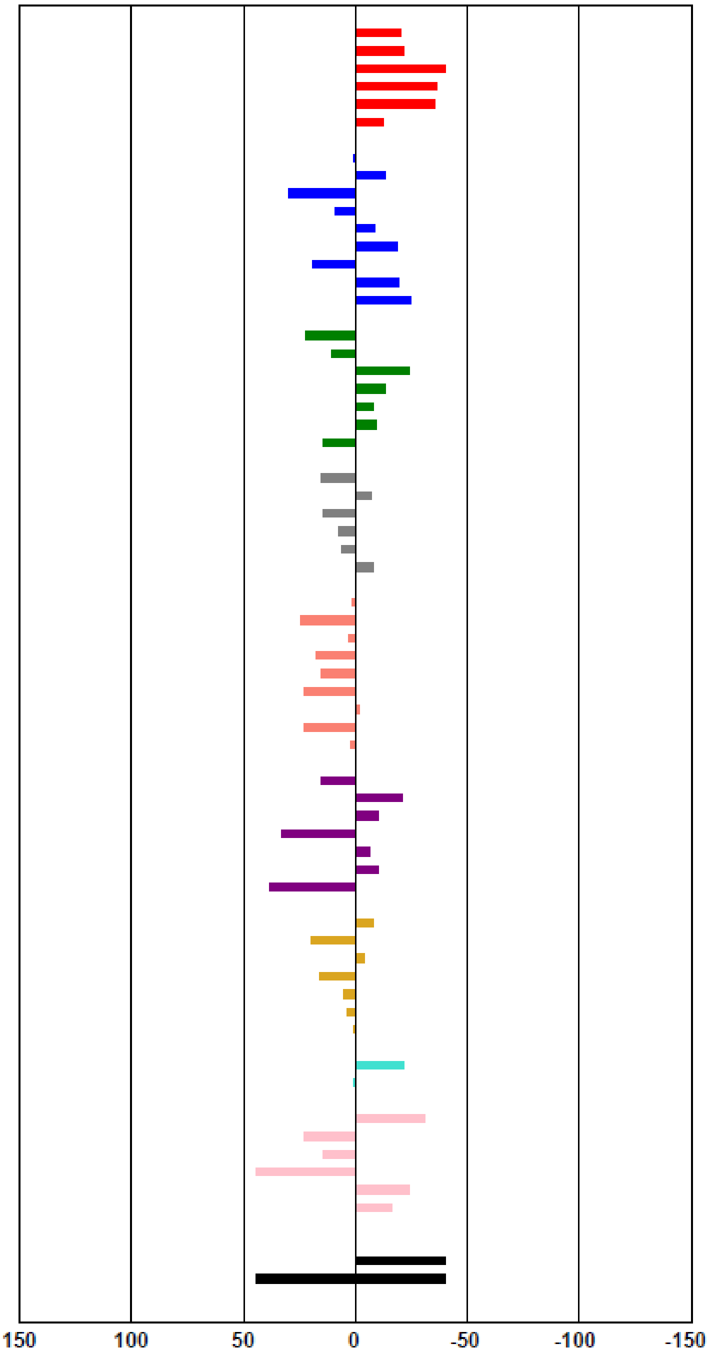

## References

1. Chen L, Fu W, Feng C, Qu R, Tong L, Zheng L, et al. Structure-based design and synthesis of 2, 4-diaminopyrimidines as EGFR L858R/T790M selective inhibitors for NSCLC. Eur J Med Chem 2017;140:510–527.

2. Guagnano V, Furet P, Spanka C, Bordas V, Le Douget M, Stamm C, et al. Discovery of 3-(2, 6-dichloro-3, 5-dimethoxy-phenyl)-1-{6-[4-(4-ethyl-piperazin-1-yl)-phenylamino]-pyrimidin-4-yl}-1-methyl-urea (NVP-BGJ398), a potent and selective inhibitor of the fibroblast growth factor receptor family of receptor tyrosine kinase. J Med Chem 2011;54(20):7066–7083.

3. Bhattarai D, Jung JH, Han S, Lee H, Oh SJ, Ko HW, et al. Design, synthesis, and biological evaluation of structurally modified isoindolinone and quinazolinone derivatives as hedgehog pathway inhibitors. Eur J Med Chem 2017;125:1036–1050.

4. El-Damasy AK, Cho NC, Nam G, Pae AN, Keum G. Discovery of a Nanomolar Multikinase Inhibitor (KST016366): A new benzothiazole derivative with remarkable broad-spectrum antiproliferative activity. ChemMedChem. 2016;11(15):1587–1595.

5. Reaction Biology Corporation. Available from: http://www.reactionbiology.com/webapps/site/Kinase_Assay_Protocol.aspx [last accessed 6 December 2022].

6. El-Damasy AK, Lee JH, Seo SH, Cho NC, Pae AN, Keum G. Design and synthesis of new potent anticancer benzothiazole amides and ureas featuring pyridylamide moiety and possessing dual B-Raf(V600E) and C-Raf kinase inhibitory activities. Eur J Med Chem 2016;115:201–216.

7. DTP Human Tumor Cell Line Screen Process: Available from: https://dtp.cancer.gov/discovery_development/nci-60/methodology.htm [last accessed 6 December 2022].

8. Tokarski JS, Newitt JA, Chang CYJ, Cheng JD, Wittekind M, Kiefer SE, et al. The structure of dasatinib (BMS-354825) bound to activated ABL kinase domain elucidates its inhibitory activity against imatinib-resistant ABL mutants. Cancer Res 2006;66(11):5790–5797.

9. Zhou TJ, Parillon L, Li F, Wang YH, Keats J, Lamore S, et al. Crystal structure of the T315I mutant of abl kinase. Chem Biol Drug Des 2007;70(3):171–181.
